# Modelling the strength of mineral–organic binding: organic molecules on the α-Al_2_O_3_(0001) surface†

**DOI:** 10.1039/d2ra04742j

**Published:** 2022-09-27

**Authors:** Aneesa Ahmad, Natalia Martsinovich

**Affiliations:** Department of Chemistry, University of Sheffield UK n.martsinovich@sheffield.ac.uk

## Abstract

Organic carbon (OC) is an essential component of soil. Sorption of OC to oxide mineral surfaces is a key process in soil preservation due to its ability to protect OC from microbial degradation. To understand the sorption of OC in soils and obtain a quantitative description of the binding of organic molecules to soil minerals, we investigated the binding of water and small organic molecules, typical building blocks of OC, on α-Al_2_O_3_, a common soil mineral. α-Al_2_O_3_ was modelled using (0001)-oriented periodic slabs, using density functional theory calculations with empirical dispersion correction. For water, dissociative adsorption was energetically preferred to molecular adsorption. Amine, amide and carboxylic acid functional groups were found to bind more strongly to this surface compared to water. Alcohol, ether, thiol and ester functional groups had adsorption energies very similar to that of water, while hydrocarbons were found to bind less strongly. Carboxylic acids were the strongest bound surface adsorbates in this study. Dissociated adsorption configurations (where allowed by the molecules' chemical nature) were usually more favourable than molecular adsorption. Hydrogen bonding was found to be a major contributor to the stability of adsorption configurations. This work shows that a number of organic functional groups, in particular amine, amide and carboxylic acids, bind to the α-Al_2_O_3_(0001) surface more strongly than water; thus they are likely to be adsorbed on this mineral surface under ambient conditions and to provide stability of adsorbed OC.

## Abstract 2

We evaluated the stability of organic molecules in soil by calculating these molecules' strength of adsorption on the α-Al_2_O_3_ mineral.

## Graphical abstract

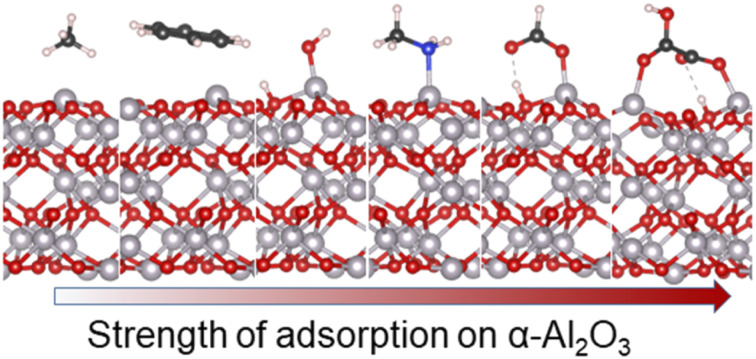

## 1. Introduction

Soil is the largest pool of organic carbon (OC) in the terrestrial biosphere.^1,2^ The presence of carbon in soil is essential for the stability and fertility of soils, as well as for storing carbon which otherwise would be released into the atmosphere as CO_2_.^3,4^ The amount of soil organic carbon (SOC) is controlled by a dynamic balance of carbon inputs (primarily through photosynthesis of plants and humification of plant litter) and carbon losses through microbial respiration which releases CO_2_ into the atmosphere, as well as through soil erosion and through leaching into groundwater.^3,5^ Humus is the largest repository of carbon in soil;^2^ it is composed of a mixture of complex organic molecules and polymers, such as polysaccharides, lignin, organic acids and disordered macromolecules (humin, humic and fulvic acids).^5–7^ Despite the relatively low content in soil by mass (typically, soils contain 0.5–6% organic carbon),^2^ SOC has a significant effect on soils' physicochemical properties: it affects the availability of nutrients, soil pH, and cation exchange capacity, dissolution of minerals and retention of water.^2,7^ However, agriculture and harvesting of crops increase the loss of SOC. Therefore, it is essential to preserve or replenish the OC in soils.^3,4^ As an important first step to achieving this, it is necessary to identify the factors that control the stability of OC in soils, and in particular the strength of binding of OC to soil mineral particles.

Sorption of OC on soil minerals is a key process that stabilizes OC and protects it from microbial decay.^8–13^ The stability of the mineral-attached OC was found to depend on several factors, such as the nature and availability of mineral surfaces during sorption, and structural properties of the OC.^8–12^ Studies of OC sorption in a variety of soils^10,14–16^ and river^17,18^ and ocean sediments^13^ showed that OC binds preferentially to aluminium and iron oxides and hydroxides. These minerals occur in soil as a result of weathering of the primary soil minerals (aluminosilicates).^5,6^ The most prominent sorption mechanism is the ligand exchange mechanism, where an organic functional group replaces a surface hydroxyl and forms a complex with metal ions (Al^3+^ or Fe^3+^) on the mineral surface; it provides strong binding and is the most efficient mechanism to stabilise OC against microbial decay.^10,11,15,18,19^

OC present in soil, such as polysaccharides and lignin, contains a variety of functional groups, *e.g.* hydroxyl, ether, amine and acid groups and saturated hydrocarbon oxane rings in polysaccharides; phenol and other aromatic groups in lignin;^5^ small organic molecules such as formic and acetic acid and amino acids are also present in soil.^20^ Spectroscopic studies have been carried out to determine the amount and the chemical nature of sorbed and unsorbed OC,^10,12,15–17,21–25^ using samples of dissolved organic matter extracted from soil or peat, which were sorbed either on samples of soil from a variety of sources^10,14,16,17,21,22^ or on specific Al or Fe oxide and hydroxide minerals, such as goethite α-FeOOH, ferrihydrite Fe_2_O_3_·*n*H_2_O, alumina α-Al_2_O_3_ and amorphous Al(OH)_3_.^12,15,21,23,24^ Distinct trends in sorption were observed depending on the nature of the organics. For all soil samples and Al and Fe minerals studied, the sorbed OC was enriched in carboxylic and aromatic groups; amino acid residues and N- and S-containing functional groups were also preferentially sorbed,^12,17^ while alkyl groups preferentially remained in water solution. The preferential sorption of carboxylic groups is consistent with the ligand exchange mechanism, while the greater affinity of aromatic than aliphatic groups towards mineral surfaces is attributed to hydrophobic interactions between aromatic groups and mineral surfaces.^15,17,21^

Thus, there is qualitative understanding of sorption of OC on soil minerals, but quantitative understanding of the mechanisms and strength of binding is lacking. In this work, we aim to obtain quantitative atomic-scale description of OC binding to soil minerals, by modelling the binding of organic molecules containing a variety of functional groups to a model mineral α-Al_2_O_3_ (corundum). α-Al_2_O_3_ is the simplest representative of the family of aluminium oxides, hydroxides and oxyhydroxides, which occurs in soil as a result of weathering of primary aluminium silicate minerals, and it has been shown to bind OC strongly.^15,23^

The structure and properties of α-Al_2_O_3_ surfaces have been extensively studied both experimentally^26–32^ and computationally.^33–39^ These studies showed that its most stable surface is the (0001)-oriented surface.^33,37–39^ The single-Al termination of this surface is the most favourable^34–39^ and is observed experimentally under ultra-high vacuum conditions.^27,28^ In the presence of water vapour, the surface is hydrated.^28,29^ Computational thermodynamics investigations showed that the preferred surface termination depends on the temperature and the partial pressure of oxygen or water vapour: the most stable termination varies from the bare surface to the fully hydroxylated surface, as the partial pressure increases.^35–37^ Multiple experimental and computational studies investigated the adsorption of water on this surface.^30–32,40–48^ Dissociative adsorption was shown to be the preferred binding mode, especially at low coverage,^30,40–47^ while complex combinations of dissociated and molecularly adsorbed water were predicted and experimentally observed at high coverages.^32,40,44,49^

There have been far fewer studies of adsorption of organic molecules onto α-Al_2_O_3_, which typically considered adsorption of one molecule or one class of molecules, *e.g.* methanol and ethanol,^50,51^ phenols,^52^ carboxylic acids,^53–55^ amines^56^ and nitro compounds.^57^ Ethanol was shown to bind slightly more strongly to the α-Al_2_O_3_(0001) surface in a dissociative configuration than in a molecular configuration.^51^ For phenols, methanol and methylamine, only non-dissociative adsorption configurations have been considered.^50,52,56^ A study of formic acid adsorption and dissociation found the 1,2-dissociated structure where the carboxyl oxygen and hydroxyl hydrogen atoms of formic acid were adsorbed, involving hydrogen bonding of the dissociated hydroxyl group, to be the most stable.^54^ An *ab initio* molecular dynamics study of pentanoic acid on α-Al_2_O_3_(0001) also found a dissociative configuration involving O⋯H hydrogen bonding.^55^ A recent study modelled adsorption of a range of organic molecules on a related mineral, γ-Al_2_O_3_, and on α-Fe_2_O_3_.^58^ Despite the importance of alumina in the environment, no comparative studies of adsorption of organic molecules on α-Al_2_O_3_-have been carried out.

Our premise is that understanding of the binding of small organic molecules on minerals (such as α-Al_2_O_3_ as a model system) is an essential first step towards understanding the binding of the more complex molecules comprising OC in soil, such as polysaccharides and lignin. Although there have been several examples of computational studies investigating the adsorption of small molecules onto the α-Al_2_O_3_(0001) surface, there has not been a comprehensive and systematic comparison of organic adsorbates. Therefore, in this work we investigate the binding of organic functional groups that are typical building blocks for OC present within soil. The small molecules under investigation are: alcohols, thiols, amines, ethers, acids, amides, esters, and hydrocarbons. We model their binding on the dry α-Al_2_O_3_(0001) surface. Although the surface under the environmental conditions is expected to be hydroxylated, the binding directly to the exposed atoms of the dry surface is a good representation of the organic molecules adsorbed *via* ligand exchange – the predominant OC sorption mechanism where organic functional groups replace surface hydroxyls to bind to metal ions of the mineral surface.^5^ We compare the binding of these organic groups to the binding of water as the most common adsorbate that covers the alumina surface under typical environmental conditions.

## 2. Computational methodology

Calculations of α-Al_2_O_3_ bulk, its (0001) surface and adsorption of molecules on this surface were performed using density functional theory (DFT) within the CP2K software package.^59–61^ The calculations utilised double-ζ basis sets with diffuse and polarization functions (DZVP) optimised for use in CP2K,^62^ and Goedecker–Teter–Hutter (GTH) pseudopotentials.^63^ CP2K uses a dual basis of atom centred Gaussian orbitals and plane waves. The plane wave cutoff energy in our calculations was 400 Ry. All calculations were done at the *Γ k*-point, since the advantage of CP2K is that it is very efficient for calculations at the *Γ* point.^60^ The rhombohedral unit cell of α-Al_2_O_3_ was used for bulk cell optimisation calculations. The angles were fixed at their experimental value *α* = *β* = *γ* = 55.28°,^64^ while the lattice constant *a* was allowed to optimise. Convergence was tested by optimising the lattice constant of multiple extended supercells (see Table S1 in the ESI†), using the PBE^65^ functional with Grimme's D3 (ref. 66) empirical dispersion correction. The 3 × 3 × 3 supercell was found to give the converged lattice constant. This supercell size was then used to compare several DFT functionals based on the generalised gradient approximation (GGA): PBE,^65^ revPBE^67^ and PBEsol^68^ functionals, all with Grimme's D3 (ref. 66) correction. The calculated lattice constants were: 5.159 Å (PBE + D3), 5.145 Å (PBEsol + D3), and 5.166 (revPBE + D3). All of these values are in good agreement with the experimental lattice constant of 5.128 Å.^64^ Thus, all of the tested GGA functionals accurately reproduce the α-Al_2_O_3_ bulk lattice parameter. The PBE functional with the D3 correction was used in all the following calculations of the α-Al_2_O_3_(0001) surface and adsorption, for easier comparison with literature studies of adsorption on this surface.

**Fig. 1:**
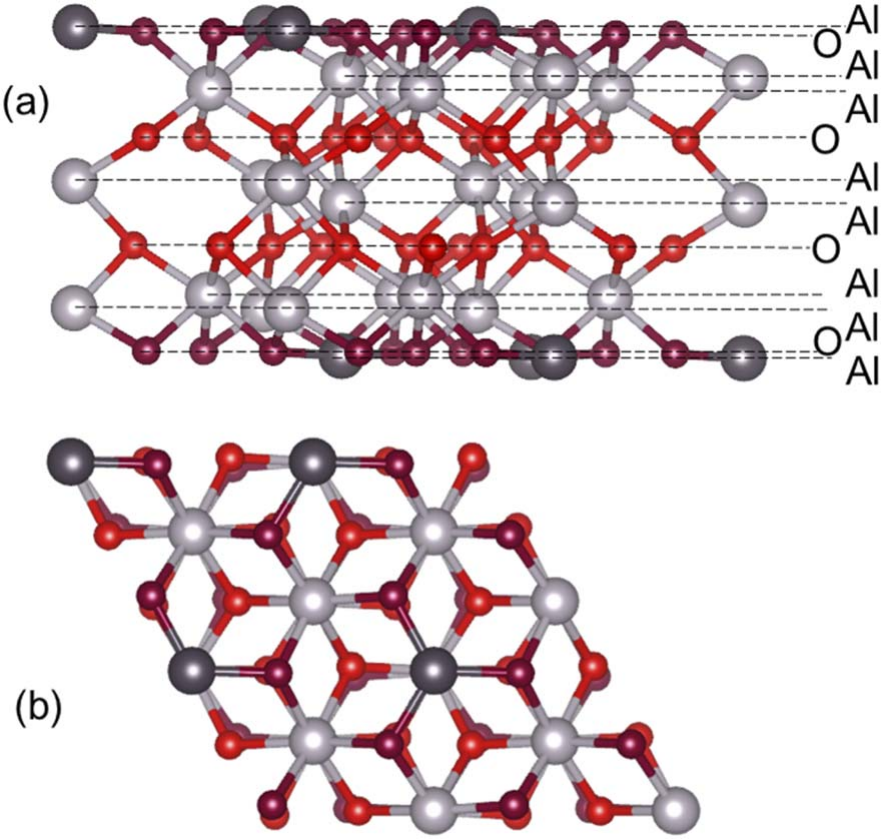
(a) Side and (b) top view of the α-Al_2_O_3_(0001) slab containing 12 atomic layers, 2 × 2 extended in the horizontal dimensions (32 Al and 48 O atoms). Surface Al atoms are shown in dark grey, subsurface O atoms in dark red, bulk Al atoms – in light grey, bulk O atoms – in red. Al and O atomic layers are indicated in the side view.

The α-Al_2_O_3_(0001) surface with the most stable single-Al termination^33–39^ (Fig. 1) was modelled using periodic slabs, where the slab lattice parameters were fixed at their optimised bulk values, while the positions of all atoms were fully optimised. Slabs were separated by 50 Å of vacuum in the vertical direction. Convergence of surface energies with respect to slab thickness and supercell size was tested. Surface energies were calculated using the equation below:1
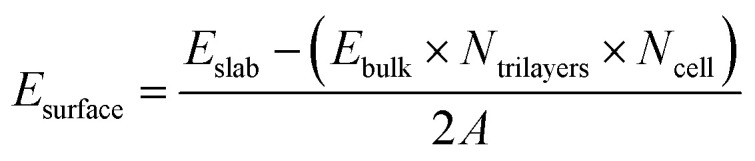
where *E*_surface_, *E*_slab_ and *E*_bulk_ are the energies of the α-Al_2_O_3_(0001) surface, α-Al_2_O_3_(0001) slab and α-Al_2_O_3_ bulk unit cell. *N*_trilayers_ is the number of Al–O–Al trilayers, *i.e.* repeat units in the slab. *N*_cell_ is the surface cell extension in the horizontal dimensions, which is 4 for the 2 × 2 extended unit cell, and 9 for the 3 × 3 extended unit cell. *A* is the surface area of the slab.

We also calculated the surface energies using an alternative approach,^69^ by fitting the slab energies to a linear function:2
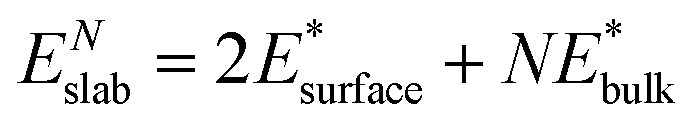
where *E*^*N*^_slab_ are the energies of slabs of thickness *N*. In this method, 
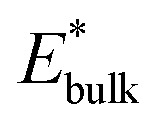
 and 
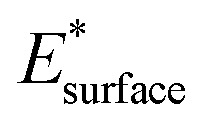
 are obtained by fitting the linear dependence of slab energies on the number of layers *N*, which gives 
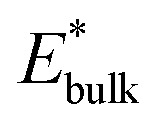
 as the slope and 
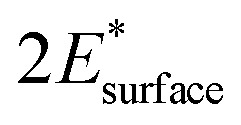
 as the intercept. This approach has the advantage of avoiding a dependence on the separately calculated *E*_bulk_ and thus avoiding a divergence in *E*_surface_, which was reported when eqn (1) is used.^69^

A converged slab size was chosen, which was then used for calculations of adsorption of water and small organic molecules. The single-Al terminated (0001) surface of α-Al_2_O_3_ exposes two types of undercoordinated atoms available for binding: surface Al atoms (shown in dark grey in Fig. 1) and O atoms (shown in dark red in Fig. 1), which are only 0.086 Å below the surface Al atoms. Adsorbates were placed on one side of the slab, and all atoms were fully optimised. Adsorption energies (*E*_ads_) were calculated as follows:

3 *E*_ads_ = *E*_slab+molecule_ − (*E*_slab_ + *E*_molecule_)

where *E*_slab+molecule_, *E*_slab_ and *E*_molecule_ are the total energies of the slab with adsorbate system, the slab and the isolated molecule, respectively. The calculated adsorption energies were corrected for the basis set superposition error (BSSE) using the counterpoise method.^70^ Mulliken charges and projected densities of states were calculated for all surface-adsorbate systems.

## 3. Results and discussion

### 3.1 Surface energies of α-Al_2_O_3_ (0001) slabs

First, we investigated the convergence of the surface energy of the single-Al terminated α-Al_2_O_3_(0001) surface with respect to the thickness of the slab and the size of the supercell. Slabs containing 6–27 atomic layers, with 2 × 2 and 3 × 3 laterally extended supercells were used. The surface energies were calculated using eqn (1) and (2) in the Computational methodology section.

The calculated surface energies of the α-Al_2_O_3_(0001)-oriented slabs are presented in Table S2 and Fig. S1.† The converged surface energy for the thickest slab with 27 atomic layers, calculated using eqn (1), is 2.04 J m^−2^ (2 × 2 supercell) or 1.97 J m^−2^ (3 × 3 supercell). The surface energy calculated using eqn (2) using all slabs is 2.00 J m^−2^ (2 × 2 supercell) or 1.99 J m^−2^ (3 × 3 supercell). These values are very close to each other, showing that the calculated surface energies are consistent with respect to the method of calculating *E*_surface_ and the supercell size. These values are also in agreement with the calculated surface energies of single-Al terminated α-Al_2_O_3_(0001) slabs reported in the literature, which range between 1.65–2.13 J m^−2^ for 9- to 18-layer slabs.^33–35,37–40^ Our converged surface energy values are slightly smaller than the experimentally determined surface energy of 2.64 J m^−2^ for α-Al_2_O_3_ crystals; however, the experimentally obtained value is believed to have contributions of higher-energy surfaces as well as the (0001) surface.^26,38^

The data in Table S2 and Fig. S1† show that the calculated surface energies change very little beyond 12 layers: *e.g.* the surface energy of the 2 × 2 extended 12-layer slab is within 0.03 J m^−2^ of the value for the thickest considered 27-layer slab, and within 0.01 J m^−2^ of the value given by eqn (2) which combines all slab energies. Therefore the surface energies of the slabs with 12 layers and above can be considered converged. The slight divergence between the surface energies calculated for 2 × 2 and 3 × 3 extended slabs can be attributed to the uncertainty in the bulk energies used in eqn (1), as described in ref. 69; however, the difference is still very small: 0.06 J m^−2^ for 27-layer slabs and 0.03 J m^−2^ for 12-layer slabs. Therefore the 2 × 2 extended 12-layer slab shown in Fig. 1 was chosen for modelling adsorption of water and small organic molecules onto the α-Al_2_O_3_(0001) surface, as the smallest converged slab that represents a good balance between accuracy and system size.

### 3.2 Adsorption of water on the α-Al_2_O_3_(0001) surface

Adsorption of water and small organic molecules on the α-Al_2_O_3_(0001) surface was investigated, to evaluate the strength of binding of various organic functional groups to this surface.

**Fig. 2:**
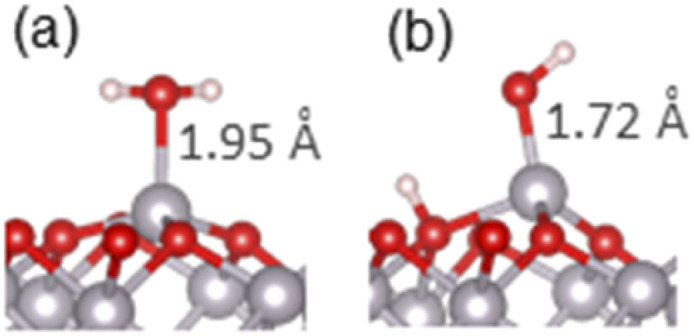
Optimised adsorption geometries of water adsorbed on the α-Al_2_O_3_(0001) surface in the (a) molecular and (b) dissociated configurations. Surface-adsorbate Al–O bond lengths are shown. In this and the following figures, light grey spheres are Al atoms, red – O atoms, white – H atoms, yellow – S atoms, blue – N atoms.

The adsorption of a water molecule on the α-Al_2_O_3_(0001) surface was investigated first. Both molecular and dissociative adsorption was considered, following the literature studies of water on α-Al_2_O_3_(0001).^40–42,44–47^ The optimised structures are shown in Fig. 2(a) and (b), and the adsorption energies are listed in Table 1. The calculated adsorption energy for the molecular adsorption of water was −1.32 eV, and the Al–O adsorption bond length was 1.95 Å, similar to the bulk Al–O distances of 1.86–1.97 Å. The dissociatively adsorbed water was more strongly bound, with the adsorption energy of −1.62 eV. The Al–O adsorption bond length was 1.72 Å, while the detached proton adsorbed on a nearby surface oxygen (the 1,2-dissociative configuration) with the O–H bond length of 0.98 Å. These Al–O bond lengths demonstrate that the water molecule is chemisorbed in both cases. The Al–O bond distances are consistent with the adsorption energies: the more strongly bound dissociated structure has a shorter chemisorption Al–O bond length.

**Table 1:** Adsorption energies and surface–adsorbate bond distances for water, methanol, methanethiol, dimethyl ether and methylamine on the α-Al_2_O_3_(0001) surface

Adsorbate	Adsorption configuration	Adsorption energy/eV	Surface–adsorbate bond distance/Å			
			Al–O	Al–S	Al–N	O–H
Water	Molecular	−1.32	1.95			
	Dissociated	−1.62	1.72			0.98
Methanol	Molecular	−1.53	1.93			
	Dissociated	−1.63	1.71			0.98
Methanethiol	Molecular	−1.36		2.40		
	Dissociated	−1.47		2.20		0.98
Dimethyl ether	Molecular	−1.66	1.92			
Methylamine	Molecular	−2.03			1.98	
	Dissociated	−1.12			1.79	0.98

The nature of adsorption can be understood more deeply by considering projected densities of states (PDOS) (Fig. S2†) and Mulliken charges on atoms (Table S3†). The Mulliken charges data for the molecularly adsorbed water show very small changes (0.02–0.04 e) in atomic charges on the surface Al and the O atom of water, indicating that the chemisorption bond in this structure is primarily covalent and there is essentially no net charge transfer. In contrast, the charge on the water oxygen in the dissociated configuration is 0.19 *e* more negative than in the molecular configuration, which explains the stronger binding of the dissociation configuration and suggests that the bonding is partly ionic. The stronger bonding is further supported by the PDOS plots (Fig. S2(a) and (b)†), which show enhanced density of states of water near the top of the valence band (VB) of the α-Al_2_O_3_ slab, and therefore an enhanced interaction of the frontier orbitals in the dissociated configuration but not in the molecular configuration.

Our calculated adsorption energies are very similar to the literature DFT values which range between −1.01 to −1.47 eV for molecular adsorption and −1.44 to −1.81 eV for dissociative adsorption of a single water molecule,^40–42,44–47^ and are also in very good agreement with the values obtained in Møller–Plesset perturbation theory calculations of water on the α-Al_2_O_3_(0001) surface: −1.31 eV for molecular and −1.61 eV for dissociative adsorption.^47^ Our adsorption bond lengths are also similar to those determined in the literature: for molecularly adsorbed water Al–O bond lengths of 1.92–1.99 Å have been reported, and for dissociatively adsorbed water, the Al–O bond lengths were between 1.70–1.75 Å, in good agreement with our results.^40–42,45,46^ These results are in agreement with experimental observations of dissociation of single water molecules on the α-Al_2_O_3_(0001) surface;^30,31^ The agreement confirms the reliability of our calculations and confirms that the 1,2-dissociative configuration is the most stable structure for an isolated water molecule adsorbed on the α-Al_2_O_3_(0001) surface.

For completeness, the adsorption of a doubly dissociated water molecule was investigated, where both hydrogen atoms were detached and bound to surface oxygen atoms. When this structure was optimised, it converged to the singly dissociated structure shown in Fig. 2(b). This provides further evidence for the favoured dissociated adsorption structure.

### 3.3 Adsorption of alcohols, thiols, amines and ethers on the α-Al_2_O_3_(0001) surface

**Fig. 3:**
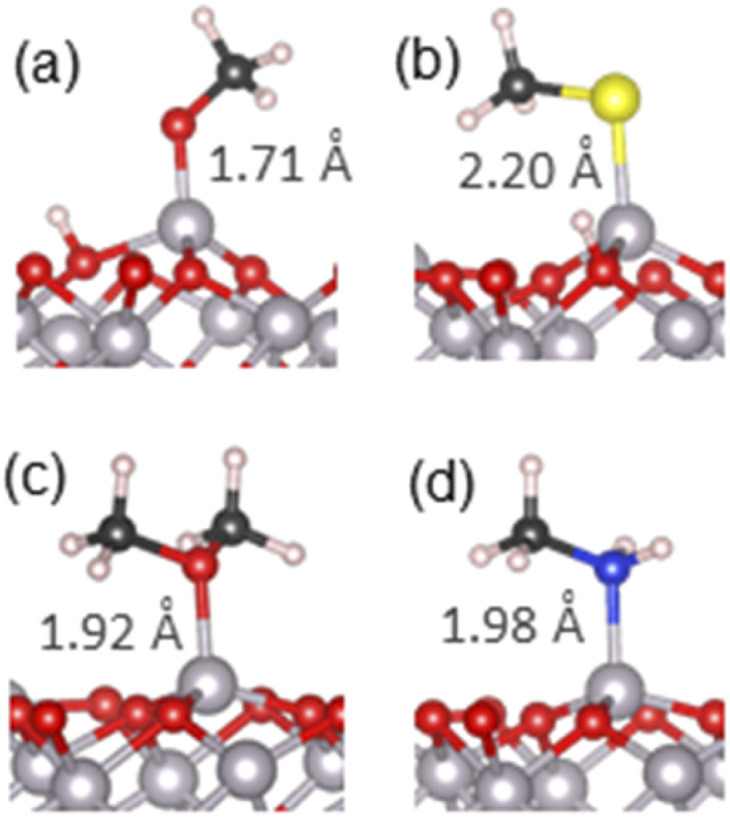
The most stable adsorption geometries of (a) methanol, (b) methanethiol, (c) dimethyl ether and (d) methylamine on the α-Al_2_O_3_(0001) surface. Surface-adsorbate bond lengths are shown.

As the next step, we modelled adsorption of small molecules containing organic functional groups which are important to carbon–soil mineral interactions. First, adsorption of alcohol, thiol and amine molecules was considered, because these groups are present in soil in polysaccharides, lignin and amino acids, and the molecules are structurally similar to water, containing a hydrogen–heteroatom (O, S or N) bond capable of dissociation. Methanol, methanethiol and methylamine were considered, as the smallest molecules containing these functional groups. Molecular and dissociative adsorption was considered for each molecule, similar to the adsorption of water. Ether group (dimethyl ether) was also considered because it is structurally similar to alcohol and is a key part of polysaccharide rings. Ethers do not have an O–H bond capable of easy dissociation, therefore only molecular adsorption of the ether was considered. The adsorption configurations for these molecules are presented in Fig. S4–S7,† and the most stable structures for each molecule are shown in Fig. 3. The adsorption energies of these structures are presented in Table 1, while the Mulliken charges are collected in Table S3,† and PDOS are plotted in Fig. S2 and S3.†

For methanol, the calculated adsorption energy for molecular adsorption was −1.53 eV, with the Al–O chemisorption bond length of 1.93 Å (structure in Fig. S4(a)†). The dissociatively adsorbed configuration (Fig. S4(b)† and detailed view in Fig. 3(a)) was slightly more strongly bound, with the adsorption energy of −1.63 eV and the much shorter Al–O chemisorption bond length of 1.71 Å; the dissociated hydrogen was bound to a nearby surface oxygen with the O–H distance of 0.98 Å. This adsorption behaviour is similar to that of water, where dissociative adsorption was also energetically favourable, and is in agreement with the previous theoretical study of ethanol on α-Al_2_O_3_(0001)^51^ which found dissociative adsorption to be ∼0.1 eV more stable than molecular adsorption. It is also consistent with the Mulliken charges of the α-Al_2_O_3_-methanol system (Table S3†), which suggest primarily covalent bonding in the molecular configuration and partly ionic bonding in the dissociative configuration, and with PDOS data (Fig. S2(c) and (d)†), which show enhanced density of the adsorbate states near the top of the valence band of α-Al_2_O_3_, and therefore stronger surface-adsorbate binding, for methanol in the dissociated configuration.

The same trend was observed for methanethiol, although with slightly weaker adsorption energies and longer bonds. The calculated adsorption energy for molecular adsorption (Fig. S5(a)†) was −1.36 eV, with the Al–S chemisorption bond length of 2.40 Å. For dissociatively adsorbed methanethiol (Fig. 3(b) and S5(b)†) the adsorption energy was −1.47 eV and the chemisorption bond lengths of the Al–S and O–H bonds were 2.20 Å and 0.98 Å. The longer Al–S bond distances compared to Al–O are attributed to the larger radius of the sulphur atom. Mulliken charges indicate some charge transfer from the adsorbate to the surface, probably originating from the diffuse orbitals of S. Similar to the adsorption of water and methanol, the PDOS plots (Fig. S2(e) and (f)†) show enhanced density of the adsorbate states near the top of the VB of α-Al_2_O_3_ for methanethiol in the dissociated configuration; however, this adsorbate's PDOS peak is smaller than in the cases of water and methanol, and there is an additional occupied state of methanethiol outside the VB of α-Al_2_O_3_, which does not contribute to interfacial bonding. This electronic structure explains the preference for the dissociated configuration, as well as the weaker adsorption of methanethiol compared to water and methanol.

For comparison with the alcohol functional group, dimethyl ether was also adsorbed onto the α-Al_2_O_3_(0001) surface in a molecular configuration (Fig. 3(c) and S6†). A dissociated structure was not considered, as it would require cleavage of the strong O–C bond. The calculated adsorption energy for molecular adsorption of dimethyl ether was −1.66 eV, slightly stronger than the adsorption of methanol, and the Al–O adsorption bond length was 1.92 Å. Very small changes in Mulliken charges on atoms at this interface confirm that the bonding is covalent. Similar to molecularly adsorbed water and methanol, there is no adsorbate's contribution at the top of the α-Al_2_O_3_ VB (Fig. S3(a)†), but the adsorbate's oxygen peak 1.75–2 eV below the VB maximum aligns with a peak in Al density of states and indicates strong Al–O interfacial bonding.

For methylamine as a model amine, the calculated adsorption energy for molecular adsorption (Fig. 3(d) and S7(a)†) was −2.03 eV, with the chemisorption bond length of 1.98 Å for the Al–N bond. For dissociatively adsorbed methylamine (Fig. S7(b)†) the adsorption energy was much weaker: −1.12 eV, and the chemisorption bond lengths for the Al–N and O–H bonds were 1.79 Å and 0.98 Å. A doubly dissociated methylamine geometry was also considered; upon optimisation this structure changed to a geometry where only one of the N–H bonds was dissociated, similar to the outcome of double dissociation of water described in the previous section. Mulliken charges indicate primarily covalent bonding in the molecular configuration and partly ionic bonding in the dissociative configuration, similar to water and its analogues. However, as seen in Fig. S3(b) and (c),† PDOS of the dissociated configuration show no good alignment of the adsorbate's states with Al peaks or with the top of the VB; in contrast, molecularly adsorbed methylamine's states align with several Al peaks in the VB, which can explain the stronger bonding of the molecular configuration of methylamine.

Comparison of these adsorption configurations shows that adsorption of thiol, alcohol and water on the α-Al_2_O_3_(0001) surface is qualitatively similar: dissociatively adsorbed configurations are energetically preferred to molecular adsorption, and the two oxygen-containing molecules (methanol and water) have very similar dissociative adsorption energies (−1.63 and −1.62 eV, respectively). Although methanol and methanethiol are isostructural, methanethiol adsorbs less strongly by ∼0.16 eV. The stronger Al–O bond can be explained by the greater electronegativity of O compared to S and therefore a greater partial negative charge of O, which results in stronger interaction with the positively charged Al. Thus, it can be concluded that the interfacial Al–S bonds are less ionic than the Al–O bonds. The Al–S bonds are also longer than the Al–O bonds; this can be attributed to the combined effect of the difference in the binding strengths and the larger radius of sulphur. Molecularly adsorbed structures are less favourable by 0.30 eV (water) or 0.10–0.11 eV (methanol and methanethiol), but they may still form, especially at the initial stages of adsorption, because molecular adsorption is likely to involve lower energy barriers than dissociative adsorption. This prediction is consistent with experimental studies of water on α-Al_2_O_3_(0001), which found dissociative adsorption to be favoured but slow under ambient conditions;^30–32^ this is also consistent with experimental studies of alcohols, which observed molecularly adsorbed methanol after adsorption on alumina at low temperatures (100–143 K),^71–73^ while adsorbed methoxide and ethoxide were observed as a result of adsorption of methanol and ethanol at room or elevated temperatures.^74,75^

Dimethyl ether can only adsorb in the molecular configuration. Comparing it to the structurally similar molecular adsorption of ethanol and water, the strength of molecular adsorption decreases in the order: dimethyl ester (−1.66 eV) > methanol (−1.53 eV) > water (−1.32 eV). This trend can be explained by the PDOS data shown in Fig. S2 and S3:† dimethyl ether's highest occupied state is the closest to the VB top of α-Al_2_O_3_ (∼2 eV below the VB top) and is the best aligned with Al-dominated states, while the highest occupied states of methanol and water are deeper in the VB (apx. 2.5 and 4.5 eV below the VB top, respectively) and are less well aligned with Al states, and therefore are less effective in facilitating strong interfacial bonding.

The most strongly bound adsorbate considered so far is molecularly adsorbed methylamine. This predicted strong adsorption is consistent with temperature programmed desorption studies,^76^ which found the maximum of methylamine desorption at 402 K (for comparison, the maximum desorption of molecularly adsorbed methanol was reported at much lower temperatures of 170–190 K or ∼250 K).^72,73^ The stronger binding of the amine compared to the alcohol can be explained by the stronger basicity of amines. Basicity can be quantified using the values of the p*K*_a_ (acidity) of the conjugate acids, *i.e.* of the protonated forms of amines and alcohols: protonated amines have a higher p*K*_a_ of ∼10 than protonated alcohols (p*K*_a_ ∼ 0),^77^ therefore amines are stronger bases than alcohols, and they have more favourable binding with surface Al^3+^ ions, which act as a Lewis acid. Methylamine is also the only adsorbate that prefers to adsorb a molecular configuration. This can be explained by the large p*K*_a_ value of a primary amine which is ∼35, compared to ∼16 for a primary alcohol and 14 for water.^77^ Therefore, the amine molecule is a very weak acid, which is unlikely to dissociate and release a proton, as the p*K*_a_ value is very high, and thus the intermediate CH_3_NH^−^ species is not stable and adsorption of non-dissociated methylamine is preferred.

**Fig. 4:**
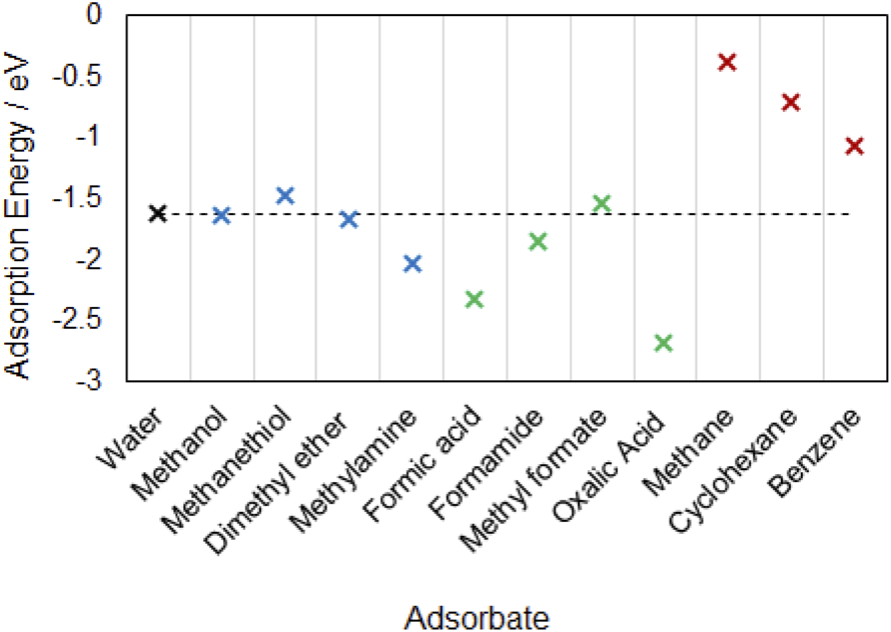
Adsorption energies of the most strongly adsorbed configurations on α-Al_2_O_3_(0001): black cross – water; blue – methanol and its analogues; green – carboxylic acid derivatives; red – hydrocarbons. The horizontal dashed line shows the adsorption energy of water in its most strongly bound dissociated adsorption configuration.

In Fig. 4 we compared the adsorption energies of these molecules in their most strongly bound configurations to the adsorption of water on α-Al_2_O_3_, since water is the most common adsorbate in soil under typical environmental conditions. Organic adsorbates with more negative adsorption energies than that of water would be energetically favourable to replace adsorbed water under ambient conditions. Notably, the molecularly adsorbed methylamine (adsorption energy of −2.03 eV) is able to bind to the α-Al_2_O_3_(0001) surface more strongly than water (adsorption energy −1.62 eV in the favoured dissociated configuration). Since the adsorption of the amine is more energetically favourable than adsorption of water, amines can be expected to displace adsorbed water molecules from this surface. Alcohol and ether groups, whose adsorption energies are very similar to that of water, are also expected to compete with water molecules for adsorption on this surface. By comparison, thiols adsorb less strongly than water and are not expected to displace water molecules from the α-Al_2_O_3_(0001) surface. Thus, organic molecules containing alcohol, ether and especially amine groups are expected to adsorb stably on this mineral surface both in the dry and water-containing environment.

### 3.4 Adsorption of acids, amides and esters on the α-Al_2_O_3_(0001) surface

**Fig. 5:**
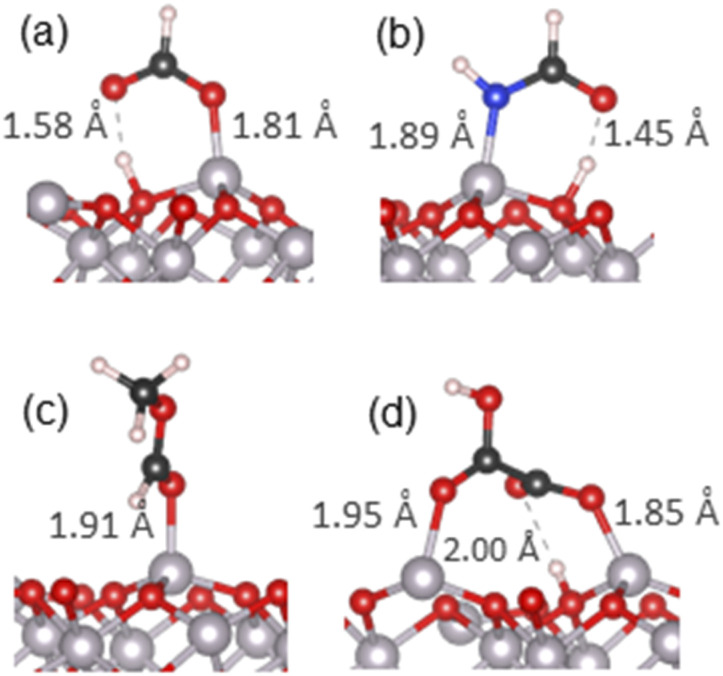
The most stable adsorption geometries of (a) formic acid, (b) formamide, (c) methyl formate and (d) oxalic acid on the α-Al_2_O_3_(0001) surface. Surface–adsorbate covalent bond and hydrogen bond lengths are shown.

Next, we consider the adsorption of carbonyl compounds: carboxylic acids, amides and esters. These functional groups are present in polysaccharides and in humic and fulvic acids which form part of OC in soil; small carboxylic acid molecules such as formic acid are also produced by microbial metabolism and exuded by plant roots.^5,20^ These functional groups are able to bind to the mineral surface *via* two atoms: the carbonyl oxygen and the second oxygen or nitrogen atom. These molecules are therefore able to adopt a great variety of possible adsorption structures. The investigated structures are presented in Table 2 and Fig. S8–S11,† and the most stable structures are summarised in Fig. 5, with their energies shown in Fig. 4.

**Table 2:** Adsorption energies and surface–adsorbate bond distances for carboxylic acid, amide and ester adsorbates on the α-Al_2_O_3_(0001) surface

Adsorbate	Adsorption configuration	Adsorption energy/eV	Surface–adsorbate bond distance/Å			
			Al–O	Al–N	O–H	O⋯H
Formic acid	Molecular, bound *via* carbonyl O	−1.42	1.92			2.23
	Molecular, bound *via* hydroxyl O	−0.95	2.01			2.34
	Dissociated	−1.80	1.76			
	Dissociated hydrogen-bonded	−2.33	1.81		1.04	1.58
Formamide	Molecular, bound *via* O	−1.64	1.87			
	Molecular, bound *via* N	−1.16		2.04		
	Dissociated, bound *via* N	−1.22		1.85	0.98	
	Dissociated hydrogen-bonded	−1.85		1.89	1.08	1.45
Methyl formate	Molecular, bound *via* carbonyl O	−1.54	1.91			2.13
	Molecular, bound *via* ether O	−1.06	2.00			
Oxalic acid	Molecular, bound *via* hydroxyl O	−1.03	2.02			
	Bridge, bound *via* carbonyl O and hydrogen bond	−1.63	1.85		1.09	1.36
	Dissociated hydrogen-bonded	−2.09	1.80		1.02	1.71
	Dissociated hydrogen-bonded bridge, bound *via* two carbonyl O	−2.69	1.85, 1.95		0.99	2.00

#### Adsorption of a carboxylic acid (formic acid)

The adsorption of formic acid as the simplest carboxylic acid was considered first. The optimised structures are presented in Fig. S8.† Two molecular adsorption configurations to the α-Al_2_O_3_(0001) surface were found possible for formic acid: adsorption through the carbonyl oxygen atom (Fig. S8(a)†) or the hydroxyl oxygen atom (Fig. S8(b)†). The calculated adsorption energy for the structure bound through the carbonyl oxygen was −1.42 eV, comparable to water and methanol molecular adsorption, and its Al–O chemisorption bond length was 1.92 Å; the shortest H⋯O (surface) distance was 2.23 Å, possibly indicating a weak surface–adsorbate hydrogen bond. For the formic acid bound through the hydroxyl oxygen the adsorption energy was −0.95 eV and its Al–O chemisorption bond length was 2.01 Å; the molecule's hydroxyl hydrogen was 2.34 Å away from the nearest surface oxygen, again possibly indicating a very weak hydrogen bond. These results show that non-dissociated formic acid prefers to bind to the α-Al_2_O_3_(0001) surface through the carbonyl oxygen; this can be attributed to easy re-arrangement of the carbonyl C=O double bond to form a single C–O bond and an interfacial Al–O bond.

Dissociative adsorption was also modelled, where one carboxylate oxygen was bound to a surface Al atom, while the dissociated hydrogen was bound to a nearby surface oxygen (Fig. S8(c)†). The adsorption energy for this configuration was −1.80 eV, with the Al–O chemisorption bond length of 1.76 Å. A dissociated chelate configuration was also considered; however, a chelate configuration was not stable and optimised instead to another dissociated structure where one of its oxygens formed a surface–adsorbate Al–O bond (1.81 Å), while the other oxygen formed an O⋯H hydrogen bond (1.58 Å) with the dissociated hydrogen that was bound to a surface oxygen (Fig. S8(d)† and a detailed view in Fig. 5(a)). Since the O–H bond of the formic acid was not fully dissociated but formed this strong hydrogen bond, we label this structure as dissociated hydrogen-bonded. The adsorption energy for this configuration was −2.33 eV, which is more favourable than the −1.80 eV adsorption energy for the fully dissociated configuration, and significantly more favourable than both molecularly adsorbed configurations. This strong binding is most likely due to the additional stabilisation due to the presence of the O⋯H hydrogen bond. The same dissociated hydrogen-bonded structure was found to have the lowest energy in an earlier computational study of formic acid adsorption by Ruan *et al.*,^54^ and is in agreement with the experimental spectroscopic observation of carboxylates adsorbed on α-Al_2_O_3_.^53^

Mulliken charges (Table S4†) show that the bonding is partly ionic and there is some charge transfer from the surface Al atoms to the adsorbate, especially in the dissociated configurations. Projected densities of states plots (Fig. S12(a)–(d)†) show that dissociatively adsorbed formic acid has states near the top of the valence band of α-Al_2_O_3_, which are well aligned with Al states, and thus this overlap of the slab and adsorbate states facilitates stronger surface–adsorbate binding. The overlap of the surface and adsorbate states is also larger than that observed for water and its analogues, which explains why the adsorption of formic acid is the strongest so far in this study.

#### Adsorption of an amide

The adsorption of formamide as a model amide was investigated. Molecular adsorption of formamide can occur through either its oxygen or nitrogen atom (Fig. S9(a) and (b)†). The oxygen-bound structure had the adsorption energy of −1.64 eV and the Al–O chemisorption bond length of 1.87 Å. The nitrogen-bound structure had the adsorption energy of −1.16 eV and the Al–N chemisorption bond length of 2.04 Å. Thus, adsorption *via* the carbonyl oxygen is more favourable, similar to the molecular adsorption of carboxylic acids. This again can be attributed to the re-arrangement of the carbonyl C=O double bond to form the single C–O bond and the interfacial Al–O bond; an additional factor is the higher electronegativity of O compared to N, which makes the Al–O bond more ionic and therefore stronger than Al–N. Moreover, unlike amines, amides are not strong bases because their lone pair is delocalised onto the carbonyl group and is less available to bind to the surface Al, compared to the strongly-binding amines. PDOS data (Fig. S12†) further support the stronger binding *via* the carbonyl O: this structure has a better alignment of the adsorbate's highest occupied state with the Al states than the nitrogen-bound structure.

Dissociative adsorption was also considered, as illustrated in Fig. S9(c), (d)† and 5(b). One N–H bond was dissociated, and the molecule was adsorbed *via* the undercoordinated nitrogen, while the dissociated hydrogen was bonded to a surface oxygen (Fig. S9(c)†). The adsorption energy of the dissociated structure was −1.22 eV, which is more favourable than molecular adsorption *via* the same nitrogen atom, but less favourable than molecular adsorption *via* the oxygen atom; the chemisorption bond lengths were 1.85 Å for the Al–N bond and 0.98 Å for the O–H bond with the surface oxygen. Finally, a dissociated chelate adsorption configuration optimised to a dissociated hydrogen-bonded configuration, similar to the case of formic acid, where the nitrogen formed a covalent bond with the surface Al, while the carbonyl oxygen formed a hydrogen bond to the dissociated hydrogen that was bonded to a surface oxygen (Fig. 5(b) and S9(d)†). This structure had the adsorption energy of −1.85 eV, and the interfacial bonds lengths of 1.89 Å (Al–N), 1.08 Å (O–H) and 1.45 Å (O⋯H) and good alignment of the adsorbate's highest occupied state with Al states near the top of the valence band. The latter structure is the most stable structure for formamide on the α-Al_2_O_3_(0001) surface.

#### Adsorption of an ester

Adsorption of methyl formate was considered as the smallest ester. Fewer configurations can be expected for esters compared to acids and amides, because there are no O–H bonds capable of easy dissociation, therefore only molecularly adsorbed configurations are possible. Optimised structures of methyl formate adsorbed on the α-Al_2_O_3_(0001) surface are shown in Fig. S10† and 5(c). The configuration in Fig. 5(c) and S10(a),† adsorbed *via* the carbonyl oxygen, had the calculated adsorption energy of −1.54 eV and the Al–O chemisorption bond length of 1.91 Å. The distance between the formate hydrogen and the nearest surface oxygen was 2.13 Å, suggesting a possible weak hydrogen bond. The configuration in Fig. S10(b),† where binding occurred through the ether oxygen of methyl formate, had the adsorption energy of −1.06 eV and the Al–O chemisorption bond length of 2.00 Å. The distance between the methyl hydrogens and the nearest surface oxygen was 2.47 Å, too long to provide additional stabilisation *via* hydrogen bonding. PDOS for adsorbed methyl formate (Fig. S13†) shows that the adsorbate's highest occupied states in both configurations are fairly deep in the α-Al_2_O_3_ VB and are not very well aligned with Al states, which explains the relatively weak binding compared to the acid and the amide.

Thus, all three types of carbonyl compounds considered so far showed adsorption *via* the carbonyl oxygen as the preferred molecularly adsorbed configuration; however, acid and amide molecules that are capable of dissociation were able to form even more strongly adsorbed dissociated hydrogen-bonded structures which involved transfer of a hydrogen atom to the surface and a hydrogen bond from the carbonyl oxygen to that hydrogen, in addition to the Al–O or Al–N covalent bond between the adsorbate and the surface.

#### Adsorption of a dicarboxylic acid

Oxalic acid, also known as 1,2-ethanedioic acid, is the simplest dicarboxylic acid, which naturally occurs in soil: it is produced by fungi and excreted by plant roots.^5^ Since oxalic acid has two carboxylic groups, a greater variety of adsorption configurations is possible than for simple carboxylic acids such as formic acid.

Molecular adsorption *via* the hydroxyl oxygen (Fig. S11(a)†) was stable, with the adsorption energy of −1.03 eV and the Al–O bond length of 2.02 Å, similar to the equivalent configuration for formic acid (Table 2). No stable configuration adsorbed only *via* the carbonyl oxygen was found; however, a more stable configuration was optimised for oxalic acid, a “bridge” structure shown in Fig. S11(b),† with the adsorption energy of −1.63 eV, which had a 1.85 Å long Al–O bond through the carbonyl oxygen and an additional hydrogen bond involving the second carboxylic group. The hydrogen of the second carboxylic group was transferred to the surface, forming a short O–H bond of 1.09 Å with the surface oxygen and retaining a bond of 1.36 Å with the carboxyl oxygen. This strong hydrogen bond accounts for the increased stability of this structure, which has a more negative adsorption energy than both the hydroxyl-bound configuration and the two molecularly adsorbed configurations of formic acid (Fig. S8(a), (b)† and Table 2). Thus, the presence of the second carboxylic group is able to provide additional bonding to the surface and therefore provide additional stability of the adsorbed structure.

Following the results for formic acid, where the dissociated hydrogen-bonded structure was the most strongly bound structure, such configurations were also considered for oxalic acid. The structure in Fig. S11(c)† is very similar to the dissociated hydrogen-bonded adsorption configuration of formic acid shown in Fig. S8(d):† the carbonyl oxygen of one of the carboxylic groups formed an Al–O chemisorption bond (1.80 Å), while a hydrogen atom of this carboxylic group was transferred to a surface oxygen but retained a hydrogen bond (1.71 Å) to the carboxylate oxygen. This structure has the adsorption energy of −2.09 eV, slightly weaker than the adsorption energy of −2.33 eV for dissociated hydrogen-bonded formic acid. Mulliken charges in the formic and oxalic acid adsorption configurations are similar, indicating that the strengths of the interfacial Al–O bonds are similar, but the shorter and therefore stronger hydrogen bond in the adsorbed formic acid (1.58 Å *vs.* 1.71 Å for oxalic acid) results in the slightly stronger adsorption of formic acid.

The most stable adsorption configuration of oxalic acid is illustrated in Fig. 5(d) and S11(d).† This is a “bridge” structure, where the oxalic acid molecule is bound to the surface *via* both carboxylic groups, with the adsorption energy of −2.69 eV. One of the carboxylic groups was not dissociated and was bound to the surface *via* the carbonyl oxygen, forming a 1.95 Å long Al–O bond. The other carboxylic group was dissociated and hydrogen-bonded: it was bound to the surface *via* the carbonyl oxygen (1.85 Å long Al–O bond), and at the same time the hydroxyl hydrogen was transferred to the surface, forming a new H–O bond but retaining a hydrogen bond (2.00 Å) to the hydroxyl oxygen. This combination of two interfacial covalent bonds and a hydrogen bond is responsible for the stability of this configuration, and is reflected in PDOS (Fig. S13(f)†), which shows that the adsorbate's multiple occupied states are very well aligned with Al states in the VB. This makes the oxalic acid dissociated “bridge” configuration the most strongly bound of all the structures explored in this study.

Our calculations showed that carboxylic acid and amide molecules prefer to adsorb in dissociated hydrogen-bonded configurations, forming both covalent bonds and hydrogen bonds to the surface. Comparison of adsorption energies shows that the acid and amide functional groups are more strongly adsorbed than water on α-Al_2_O_3_. Dicarboxylic acids are able to bind particularly strongly, by forming “bridge” structures where both carboxylic groups bind to the surface. Thus, we can conclude that acid and amide molecules would be able to displace water when binding to α-Al_2_O_3_ in soils because they bind more strongly than water, specifically in their dissociated hydrogen-bonded configurations. The ester functional group is able to adsorb onto the surface only in molecular configurations. The strongest adsorption energy for the ester is slightly (by 0.08 eV) weaker than the adsorption energy of water. Therefore, esters are not very likely to displace water from the α-Al_2_O_3_(0001) surface, but they may still compete with water for adsorption sites.

### 3.5 Adsorption of hydrocarbons on the α-Al_2_O_3_(0001) surface

Organic molecules that form in soil through decomposition of plants (*e.g.* cellulose and other polysaccharides) typically contain sp^3^ hybridised carbons as well as functional groups. Sorption studies also indicated presence of aromatic groups in OC sorbed in soils, *e.g.* lignin.^5,10,15,17,21–24^ Therefore, adsorption of simple aliphatic and aromatic hydrocarbons was modelled (Fig. S14–S16† and Table 3), to compare them to the adsorption of functional groups. Methane was considered as the simplest example of a hydrocarbon molecule. Cyclohexane and benzene were modelled as comparable examples of aliphatic and aromatic hydrocarbons containing the same numbers of carbon atoms. Cyclohexane is particularly relevant because if can be viewed as an analogue of tetrahydropyran (with an oxygen atom replaced by a CH_2_ group), the 6-membered ring that is the building block of polysaccharides. Only molecular adsorption was considered for these molecules, since dissociation of hydrocarbons is not likely under normal environmental conditions. Mulliken charges (Table S5†) and projected densities of states (Fig. S17†) were analysed, to understand the nature of bonding.

**Table 3:** Adsorption energies and surface–adsorbate bond distances for hydrocarbon adsorbates on the α-Al_2_O_3_(0001) surface

Adsorbate	Adsorption configuration	Adsorption energy/eV	Surface–adsorbate distance/Å	
			Al–C	Al–H
Methane	Molecular	−0.38	2.48	2.13, 2.21
Cyclohexane	Molecular (tilted)	−0.71	2.43	2.01, 2.09
	Molecular (horizontal)	−0.64	2.93	1.93
Benzene	Molecular	−1.08	2.27	

Methane adsorbed on α-Al_2_O_3_(0001) with the adsorption energy of −0.38 eV, with the Al–C distance of 2.48 Å and the Al–H shortest distances of 2.13 and 2.21 Å (Fig. S14†), which are longer than the sums of the atoms' covalent radii (2.20 Å for Al–C and 1.80 Å for Al–H).^78^ Changes in Mulliken charges are also very small, indicating no significant charge rearrangement, and the surface and adsorbate's states in the PDOS are not aligned, showing lack of covalent bonding. This binding is clearly much weaker than water or any organic functional groups and is attributed to van der Waals dispersion interactions.

Cyclohexane adsorption was modelled with two initial positions, both parallel to the surface, with the centre of the ring above a surface O or above a surface Al atom. As a result of optimisation, these adsorption positions changed to (a) a tilted geometry with the centre of the molecule above a subsurface oxygen (Fig. S15(a)) and (b)† a horizontal geometry with the centre of the molecule above a surface oxygen (Fig. S15(b)†). Both structures had fairly similar adsorption energies: −0.71 and −0.64 eV, respectively (−0.12 and −0.11 eV per carbon atom). The shortest Al–C distances were 2.43 Å (structure (a)) and 2.93 Å (structure (b)), while the shortest Al–H distances were between 1.93–2.09 Å, longer than the sum of the atoms' covalent radii. Similar to methane, these distances and adsorption energies, as well as the lack of charge transfer and lack of interaction in the PDOS, indicate that the adsorption is due to van der Waals dispersion interactions.

Finally, benzene was adsorbed as the simplest aromatic hydrocarbon. Similar to cyclohexane, two initial positions parallel to the surface were considered, with the centre of the ring above a surface Al or a surface O atom. Both calculations resulted in the same optimised adsorption structure (Fig. S16†), where one of the benzene carbon atoms is above a surface aluminium atom with the Al–C distance of 2.27 Å, which is only slightly longer than the sum of the atoms' covalent radii (2.20 Å) and suggests that the interaction may be partly covalent in character. This interpretation is supported by PDOS (Fig. S17(d)),† where some of the adsorbate's states are aligned with some of the α-Al_2_O_3_ states, unlike in the aliphatic hydrocarbons, and with Mulliken charges (Table S5†) suggesting redistribution of charges in the benzene molecule. The adsorption energy was −1.08 eV (−0.18 eV per C atom), more negative than for cyclohexane, indicating that aromatic hydrocarbons adsorb more strongly than aliphatic hydrocarbons on the α-Al_2_O_3_(0001) surface.

The adsorption energies of methane, cyclohexane and benzene are all much weaker than those of water and of organic functional groups. We can therefore conclude that they will not displace water when binding to the α-Al_2_O_3_(0001) surface in soils because water binds more strongly. Moreover, since organic functional groups bind more strongly than hydrocarbons, these functional groups are likely to be the major factors controlling the binding of OC to soil minerals. However, our calculations show that the binding of hydrocarbons is non-negligible and will contribute significantly to the overall binding of OC to soils; notably, the binding of aromatic hydrocarbons is more favourable than that of aliphatic hydrocarbons, suggesting that aromatic hydrocarbons are more likely to be preserved in soil.

## 4. Conclusions

In this investigation, we obtained a quantitative description of the binding of organic molecules to a model soil mineral, α-Al_2_O_3_ (corundum), which is associated with sorption of organic carbon in soil.^21^ The binding of water and small organic molecules (alcohols, thiols, amines, ethers, acids, esters, amides and hydrocarbons) in a variety of adsorption configurations on the α-Al_2_O_3_(0001) surface was investigated. The adsorption energies were compared to determine which functional groups bind to the α-Al_2_O_3_(0001) surface most strongly, and thus to evaluate their ability to replace water hydroxyl groups which cover this mineral surface under ambient conditions. Organic molecules containing such strongly bound groups are expected to be stable in soils.

Alcohols, thiols, amines, acids and amides were able to adsorb strongly, forming interfacial covalent bonds. Alcohols, thiols, acids and amides adsorbed more strongly in dissociated configurations, while amines, ethers and esters adsorbed more strongly in molecular configurations. For acids and amides, the most stable adsorption configurations were dissociated configurations that were stabilised by hydrogen bonding with the dissociated proton.

Carboxylic acids and amine were the strongest bound adsorbates in this study; they were found to bind to the α-Al_2_O_3_(0001) surface considerably more strongly than water, as shown in the summary of adsorption energies in Fig. 4. Therefore, we expect that these functional groups will displace water when adsorbing on this mineral surface.

A number of functional groups were found to have the adsorption energies quite close to that of water, *e.g.* methanol and dimethyl ether have their strongest adsorption energies very similar to that of water; formamide is able to bind slightly more strongly than water, while methyl formate and methanethiol in their most strongly adsorbed configurations are slightly less strongly adsorbed than water. Therefore we expect that these groups, especially amides, ethers and alcohols, can compete with water in binding to the α-Al_2_O_3_(0001) surface.

In contract, the binding of hydrocarbons on α-Al_2_O_3_(0001) occurred *via* dispersion interactions and was considerably weaker than the binding of water. The aromatic hydrocarbon benzene was found to bind more strongly that its aliphatic counterpart, cyclohexane; however, neither of them could bind strongly enough to be able to displace adsorbed water from the α-Al_2_O_3_(0001) surface. Therefore, we conclude that strong sorption of organic molecules in soil requires organic functional groups, such as acids or amines.

Our observed stabilities of adsorption of different functional groups agree with the results of experimental soil chemistry studies, which reported preferential adsorption of carboxylic groups,^10,15,17,21–24^ amino acid residues and other O, N and S-containing groups in soil.^16,17,25^ Our results are also consistent with the ligand exchange mechanism of OC binding: the energies summarised in Fig. 4 show that replacement of surface hydroxyls (*i.e.* adsorbed dissociated water) with strongly binding organic groups such as acids and amines is thermodynamically favourable. Our finding of the stronger adsorption of aromatic than aliphatic hydrocarbons is also consistent with the experiments finding that aromatic carbon preferentially sorbs in soil and on soil minerals, while aliphatic carbon accumulates in solution.^10,15,17,21–24^

Thus, our investigation has determined the key functional groups, such as carboxylic acid, amine and aromatic groups, which are essential for strong binding of organic molecules to the α-Al_2_O_3_(0001) surface. These findings will help to develop the understanding of adsorption of large biomolecules, such as polysaccharides and lignin, on α-Al_2_O_3_ and other minerals, to obtain a more realistic representation of the binding of OC to soil minerals.

A limitation of this work is that only the clean α-Al_2_O_3_(0001) surface was considered, and the effect of the environment was included only by considering the competition with adsorbed dissociated water molecules. It is known that the α-Al_2_O_3_(0001) surface is hydroxylated under environmental conditions;^28,29,32,35–37^ moreover, aluminium oxyhydroxide (gibbsite Al(OH)_3_) is more widespread in soil than aluminium oxide.^5^ Therefore, as the next step, adsorption on other mineral surfaces, such as the hydroxylated α-Al_2_O_3_(0001) surface, will need to be considered as a more realistic environment for soil minerals, and the activation barriers for the exchange of surface hydroxyls for organic adsorbates will need to be modelled.

## Data availability

The data underlying this study (optimised structures of all adsorption configurations) are openly available in the University of Sheffield ORDA (online research data) repository at https://doi.org/10.15131/shef.data.19486907.

## Author contributions

The manuscript was written through contributions of all authors.

## Conflicts of interest

There are no conflicts to declare.

## Supplementary Material

RA-012-D2RA04742J-s001
