# Supplementary material for: Modelling the strength of mineral–organic binding: organic molecules on the α-Al2O3(0001) surface
Source: RSC Adv. 2022 Sep 27;12(42):27604–15. doi: 10.1039/d2ra04742j (PMC9514380; doi:10.1039/d2ra04742j)
Supplement: RA-012-D2RA04742J-s001 [file RA-012-D2RA04742J-s001.pdf]

Electronic Supplementary Material (ESI) for RSC Advances.
This journal is © The Royal Society of Chemistry 2022

Electronic Supplementary Information for

Modelling the Strength of Mineral-Organic Binding: Organic Molecules on the α-Al_2O_3 (0001) Surface

Aneesa Ahmad, Natalia Martsinovich*
Department of Chemistry, University of Sheffield, UK

Table S1. Values of the optimised lattice parameter *a* of the rhombohedral α-Al_2O_3 bulk unit cell, calculated using different supercell sizes and different density functionals.

Table S2. Surface energies (J m^{-2}) of α-Al_2O_3 (0001)-oriented slabs of different thicknesses.

Figure S1. Dependence of the surface energies on the slab thickness for α-Al_2O_3 (0001)-oriented slabs.

Table S3. Mulliken charges on Al atoms of the α-Al_2O_3 slabs and on water, methanol, methanetiol, dimethyl ether and methylamine adsorbates' O, S and N atoms involved in chemisorption.

Figure S2. Projected densities of states (PDOS) for α-Al_2O_3 (0001) slabs with adsorbates: water (a) molecularly adsorbed and (b) dissociatively adsorbed; methanol (c) molecularly adsorbed and (b) dissociatively adsorbed; methanethiol: (e) molecularly adsorbed and (f) dissociatively adsorbed. In this and the following PDOS plots, black lines show contributions of Al, red – O of α-Al_2O_3, pink – O of adsorbate, blue – H, green – C, orange -S, cyan – N.

Figure S3. Projected densities of states (PDOS) for α-Al_2O_3 (0001) slabs with adsorbates: (a) dimethyl ether molecularly adsorbed; methylamine (b) molecularly adsorbed and (c) dissociatively adsorbed.

Figure S4. Optimised adsorption geometries of methanol adsorbed on the α-Al_2O_3 (0001) surface in the (a) molecular and (b) dissociated configurations. In this and the following figures, light grey spheres are Al atoms, red – O atoms, white – H atoms, yellow – S atoms, blue – N atoms.

Figure S5. Optimised adsorption geometries of methanethiol adsorbed on the α-Al_2O_3 (0001) surface in the (a) molecular and (b) dissociated configurations.

Figure S46. Optimised adsorption geometry of dimethyl ether adsorbed on the α-Al_2O_3 (0001) surface in the molecular configuration.

Figure S7. Optimised adsorption geometries of methylamine adsorbed on the α-Al_2O_3 (0001) surface in the (a) molecular and (b) dissociated configurations.

Figure S8. Optimised adsorption geometries of formic acid adsorbed on the α-Al_2O_3 (0001) surface. Panels (a) and (b) show molecular adsorption configurations: (a) adsorption via the carbonyl oxygen of formic acid and (b) adsorption via the hydroxyl oxygen of formic acid. Panel (c) shows the dissociated configuration, and panel (d) shows the dissociated hydrogen-bonded configuration.

Figure S9. Optimised adsorption geometries of formamide adsorbed on the α-Al_2O_3 (0001) surface. Panels (a) and (b) show molecular configurations: (a) adsorption via the oxygen atom and (b) adsorption via the nitrogen atom. Panel (c) shows the dissociated configuration, and panel (d) shows the dissociated hydrogen-bonded configuration.

Figure S10. Optimised adsorption geometries of methyl formate adsorbed on the α-Al_2O_3 (0001) surface in two molecularly adsorbed configurations: (a) adsorption via the carbonyl oxygen and (b) adsorption via the ether oxygen of methyl formate.

Figure S11. Optimised adsorption geometries of oxalic acid on the α-Al_2O_3 (0001) surface: (a) molecular adsorption via hydroxyl oxygen, (b) bridge via carbonyl oxygen and hydrogen bond, (c) dissociated with hydrogen bond and (d) dissociated bridge via two carbonyl oxygens and hydrogen bond.

Table S4. Mulliken charges on Al atoms of the α-Al_2O_3 slabs and on formic acid, formamide, methyl formate and oxalic acid adsorbates' O and N atoms involved in chemisorption.

Figure S12. Projected densities of states for α-Al_2O_3 (0001) slabs with adsorbates: formic acid (a) molecularly adsorbed via the carbonyl oxygen, (b) molecularly adsorbed via the hydroxyl oxygen, (c) dissociatively adsorbed, (d) dissociatively adsorbed with hydrogen bonding; formamide (e) molecularly adsorbed via the carbonyl oxygen, (f) molecularly adsorbed via the nitrogen, (g) dissociatively adsorbed, (h) dissociatively adsorbed with hydrogen bonding.

Figure S13. Projected densities of states for α-Al_2O_3 (0001) slabs with adsorbates: (a) methyl formate adsorbed via the carbonyl oxygen, (b) methyl formate adsorbed via the ether oxygen, (c) oxalic acid adsorbed via a hydroxyl oxygen, (d) oxalic acid in the bridge geometry adsorbed via a carbonyl oxygen and a hydrogen bond, (e) oxalic acid in the dissociated hydrogen-bonded geometry, (f) oxalic acid in the dissociated hydrogen-bonded bridge geometry, adsorbed via two carbonyl oxygen.

Figure S14. Optimised adsorption geometry of methane adsorbed on the α-Al_2O_3 (0001) surface: (left) side and (right) top view.

Figure S15. Optimised adsorption geometries of cyclohexane on the α-Al_2O_3 (0001) surface: (a) tilted geometry (side and top view), and (b) horizontal geometry (side and top view).

Figure S16. Optimised adsorption geometry of benzene on the α-Al_2O_3 (0001) surface: (left) side and (right) top view.

Table S5. Mulliken charges on Al atoms of the α-Al_2O_3 slabs closest to the adsorbate and the adsorbed methane, cyclohexane and benzene carbon atoms closest to the surface.

Figure S17. Projected densities of states for α-Al_2O_3 (0001) slabs with adsorbates: (a) methane, (b) cyclohexane in the tilted geometry, (c) cyclohexane in the horizontal geometry, and (d) benzene.

Table S1. Values of the optimised lattice parameter *a* of the rhombohedral α-Al_2O_3 bulk unit cell, calculated using different supercell sizes and different density functionals.

DFT functional	Bulk supercell size	*a*, Å
PBE[1] + D3[2]	1×1×1	5.143
PBE[1] + D3[2]	2×2×2	5.158
PBE[1] + D3[2]	3×3×3	5.159
PBE[1] + D3[2]	4×4×4	5.159
revPBE[3] + D3[2]	3×3×3	5.145
PBEsol[4] + D3[2]	3×3×3	5.166
Experiment[5]	N/A	5.128

Table S2. Surface energies (J m^{-2}) of α-Al_2O_3 (0001)-oriented slabs of different thicknesses.

No. of atomic layers	Surface Energy / J m^{-2}	
	2×2 extended cell	3×3 extended cell
6 [a]	2.097	2.070
9 [a]	1.899	1.875
12 [a]	2.006	1.975
15 [a]	2.028	1.984
18 [a]	2.026	1.980
21 [a]	2.029	1.976
24 [a]	2.038	1.978
27 [a]	2.036	1.969
6 – 27 [b]	1.998	1.989

[a] Calculated using equation (1) in the main text.

[b] Calculated using equation (2) in the main text.

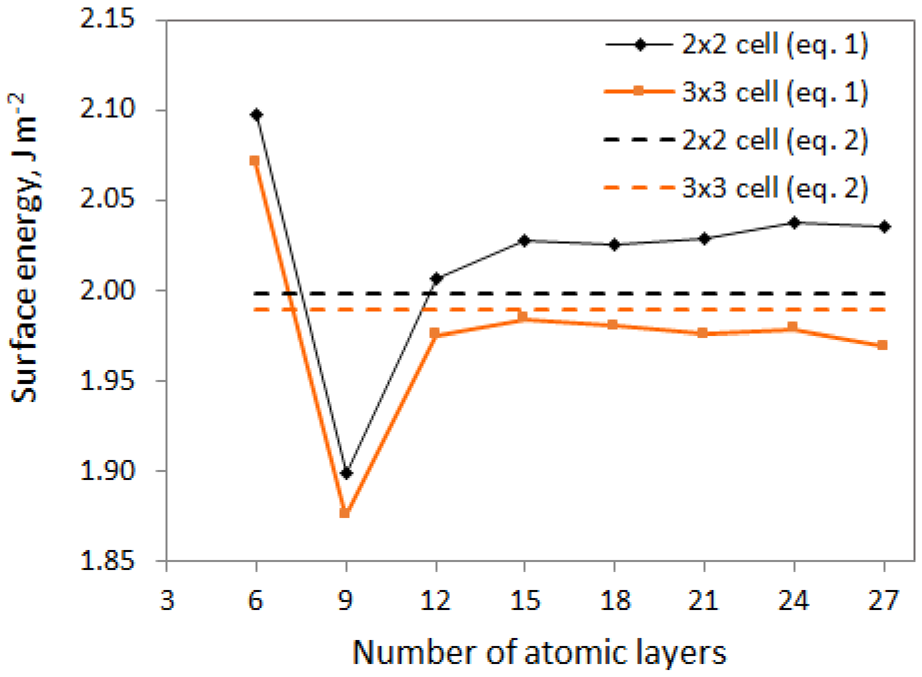

Figure S1. Dependence of the surface energies on the slab thickness for α-Al_2O_3 (0001)-oriented slabs, for two supercell sizes. The surface energies calculated using equation (1) in the main text are shown as solid lines with symbols. The surface energies calculated using equation (2) in the main text by combining the energies of all slabs (6 – 27 layers) are shown as horizontal dashed lines.

Table S3. Mulliken charges on Al atoms of the α-Al_2O_3 slabs and on water, methanol, methanetiol, dimethyl ether and methylamine adsorbates' O, S and N atoms involved in chemisorption.

**Adsorbate**	**Adsorption configuration**	**Charges on surface Al**			**Charges on adsorbates' O/S/N**		
		**before adsorption**	**after adsorption**	**charge difference**	**before adsorption**	**after adsorption**	**charge difference**
Water	molecular	1.23	1.25	0.02	-0.39	-0.35	0.04
	dissociated	1.23	1.29	0.07	-0.39	-0.54	-0.15
Methanol	molecular	1.23	1.28	0.05	-0.32	-0.35	-0.03
	dissociated	1.23	1.33	0.10	-0.32	-0.49	-0.17
Methanethiol	molecular	1.23	1.01	-0.21	0.09	0.28	0.19
	dissociated	1.23	0.95	-0.28	0.09	-0.02	-0.11
Dimethyl ether	molecular	1.23	1.30	0.08	-0.25	-0.34	-0.09
Methylamine	molecular	1.23	1.22	-0.01	-0.19	-0.19	-0.01
	dissociated	1.23	1.19	-0.04	-0.19	-0.35	-0.16

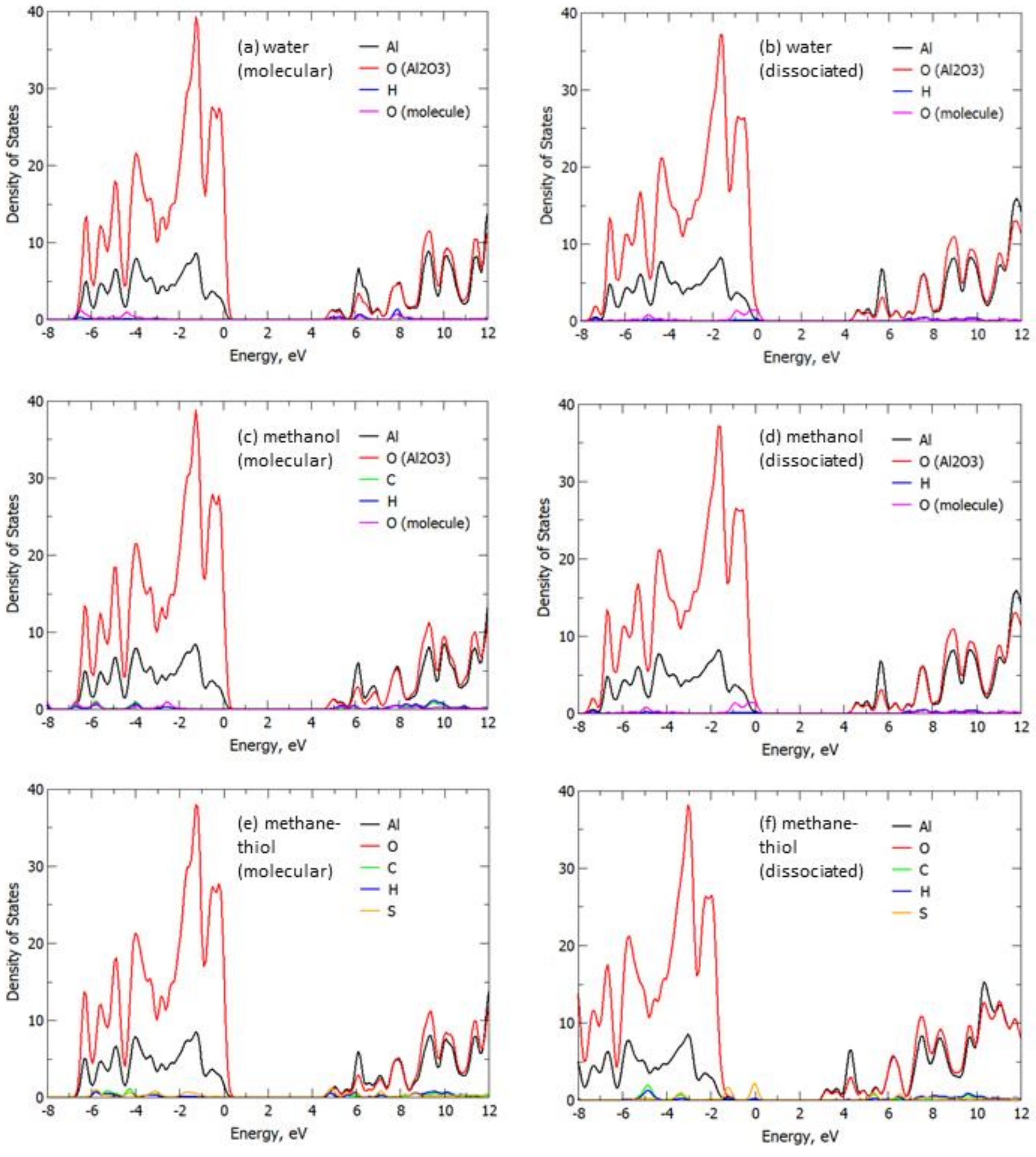

Figure S2. Projected densities of states (PDOS) for α-Al_2O_3 (0001) slabs with adsorbates: water (a) molecularly adsorbed and (b) dissociatively adsorbed; methanol (c) molecularly adsorbed and (b) dissociatively adsorbed; methanethiol: (e) molecularly adsorbed and (f) dissociatively adsorbed. In this and the following PDOS plots, black lines show contributions of Al, red – O of α-Al_2O_3, pink – O of adsorbate, blue – H, green – C, orange -S, cyan – N.

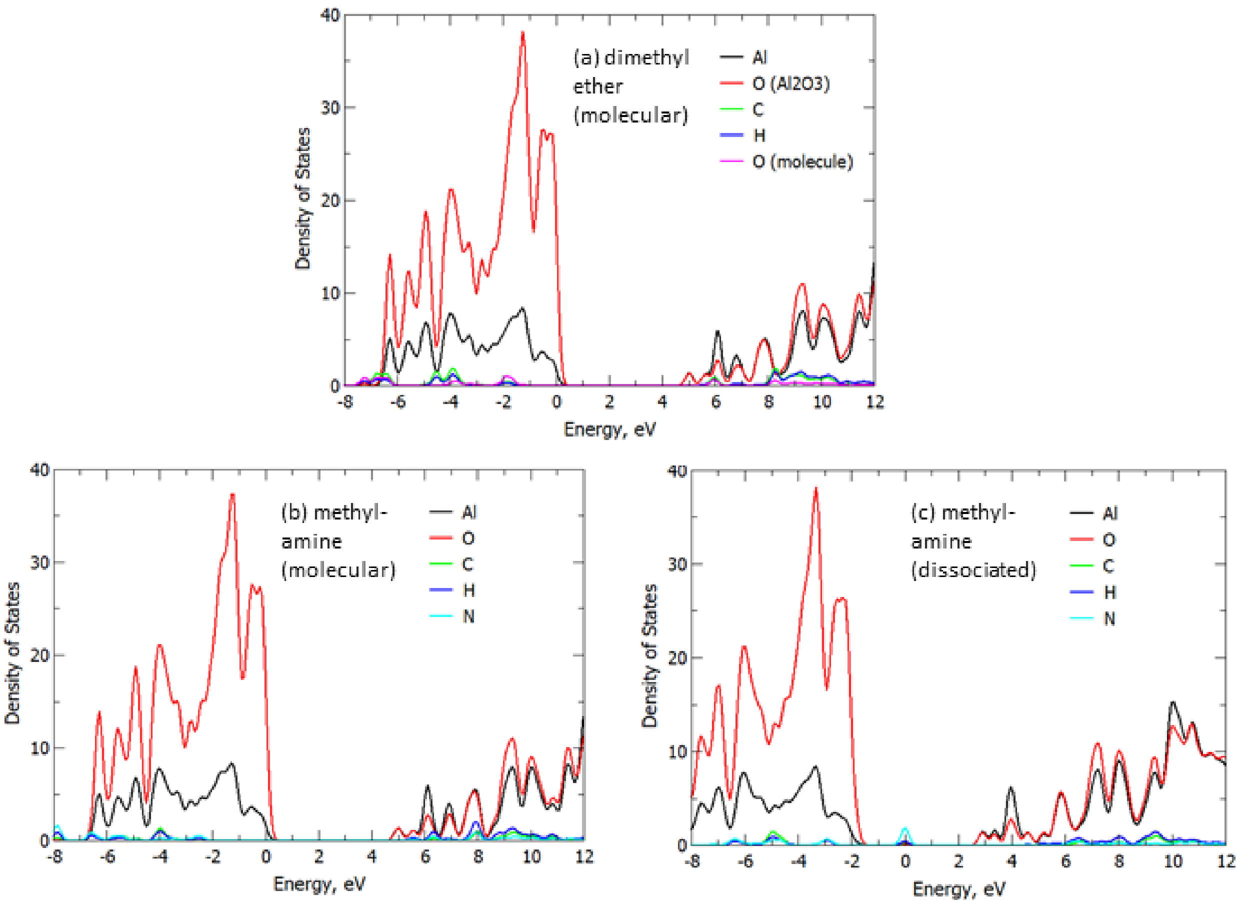

Figure S3. Projected densities of states (PDOS) for α-Al_2O_3 (0001) slabs with adsorbates: (a) dimethyl ether molecularly adsorbed; methylamine (b) molecularly adsorbed and (c) dissociatively adsorbed.

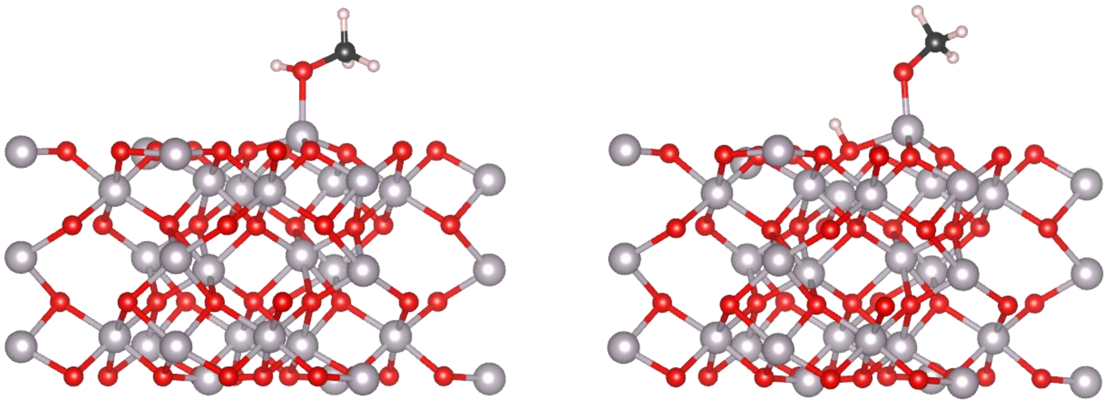

(a) (b)

Figure S4. Optimised adsorption geometries of methanol adsorbed on the α-Al_2O_3 (0001) surface in the (a) molecular and (b) dissociated configurations. In this and the following figures, light grey spheres are Al atoms, red – O atoms, white – H atoms, yellow – S atoms, blue – N atoms.

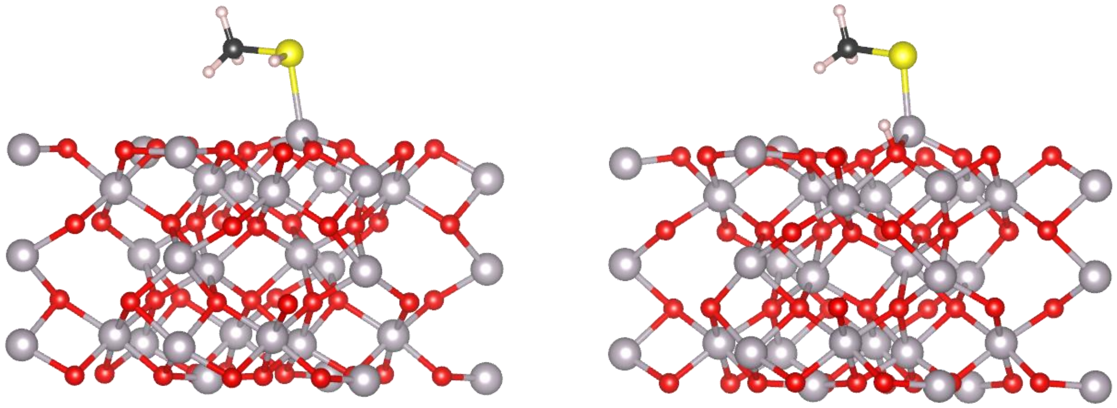

(a) (b)

Figure S5. Optimised adsorption geometries of methanethiol adsorbed on the α-Al_2O_3 (0001) surface in the (a) molecular and (b) dissociated configurations.

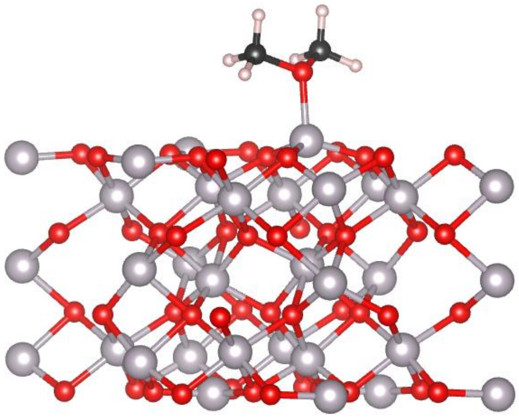

Figure S6. Optimised adsorption geometry of dimethyl ether adsorbed on the α-Al_2O_3 (0001) surface in the molecular configuration.

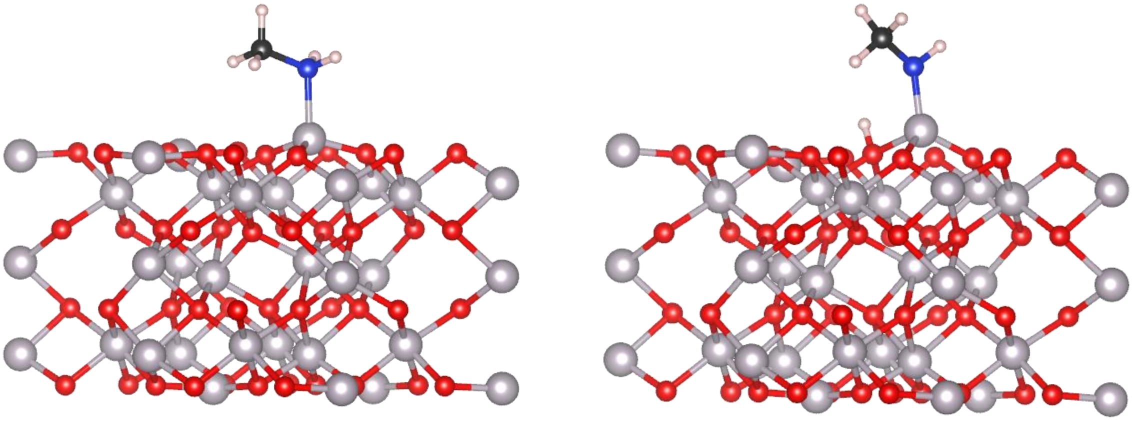

(a) (b)

Figure S7. Optimised adsorption geometries of methylamine adsorbed on the α-Al_2O_3 (0001) surface in the (a) molecular and (b) dissociated configurations.

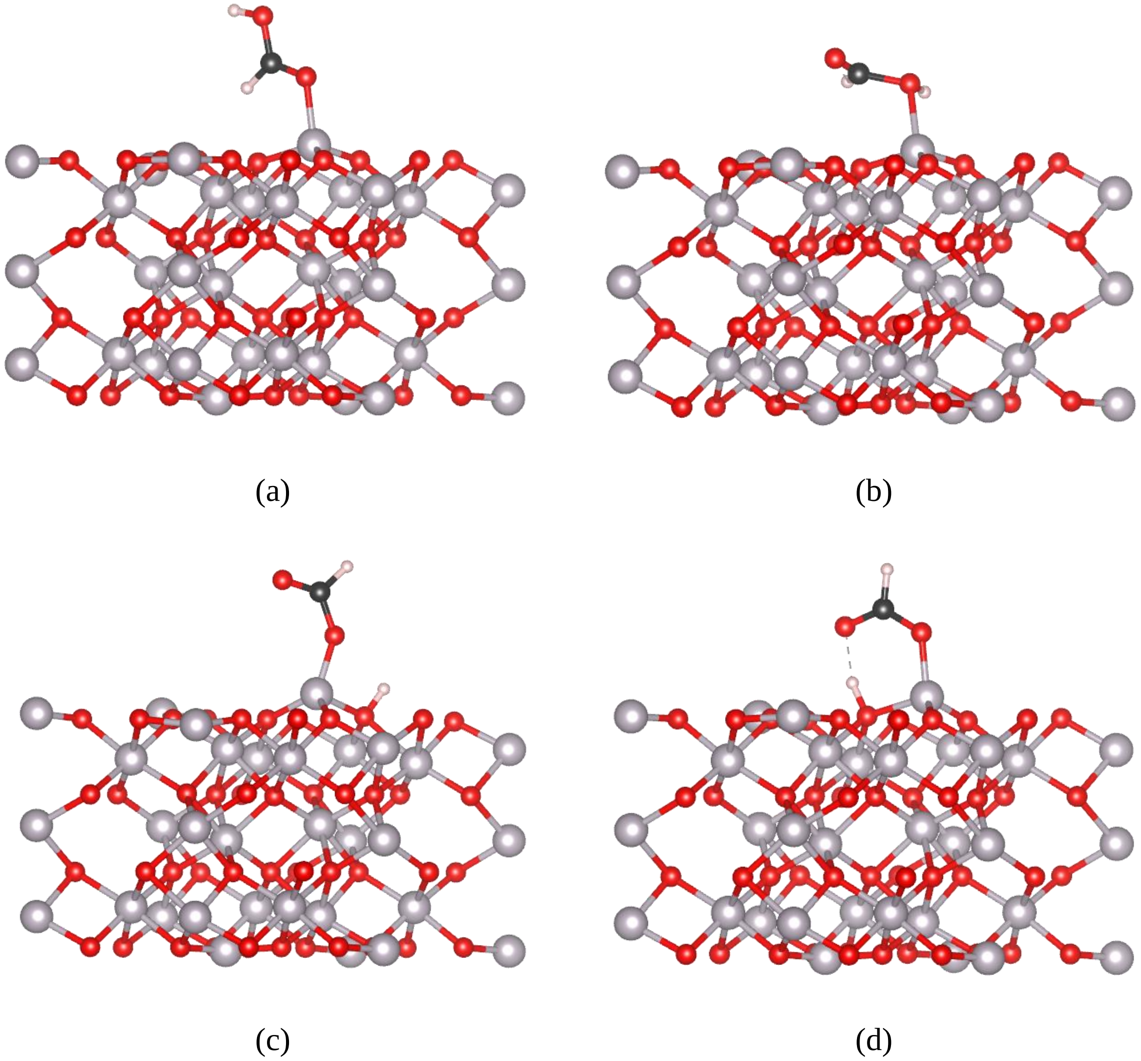

Figure S8. Optimised adsorption geometries of formic acid adsorbed on the α-Al_2O_3 (0001) surface. Panels (a) and (b) show molecular adsorption configurations: (a) adsorption via the carbonyl oxygen of formic acid and (b) adsorption via the hydroxyl oxygen of formic acid. Panel (c) shows the dissociated configuration, and panel (d) shows the dissociated hydrogen-bonded configuration.

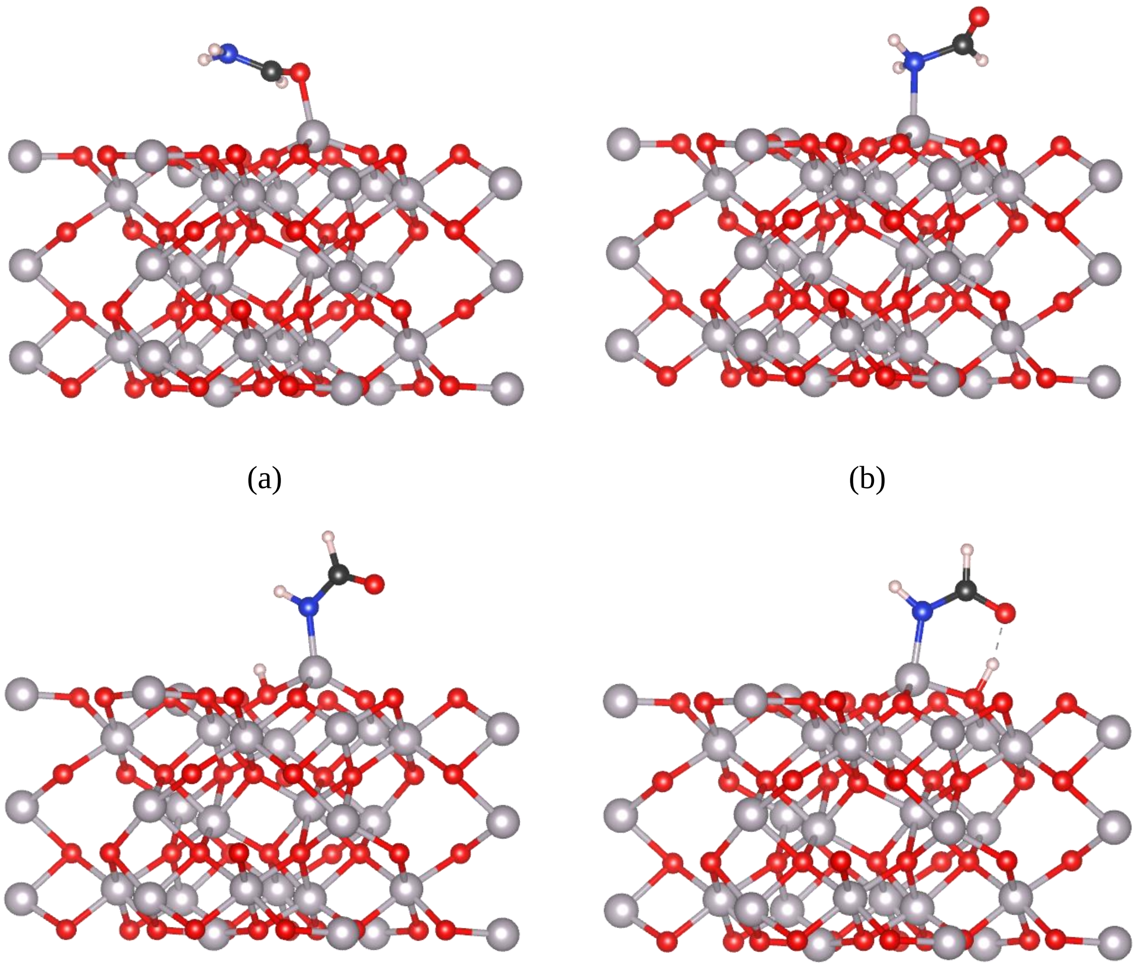

(a) (b)

(c) (d)

Figure S9. Optimised adsorption geometries of formamide adsorbed on the α-Al_2O_3 (0001) surface. Panels (a) and (b) show molecular configurations: (a) adsorption via the oxygen atom and (b) adsorption via the nitrogen atom. Panel (c) shows the dissociated configuration, and panel (d) shows the dissociated hydrogen-bonded configuration.

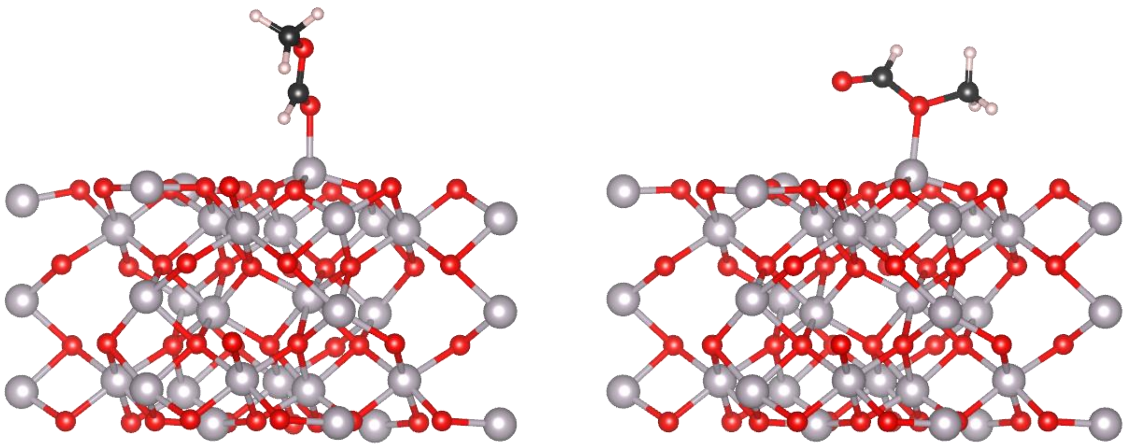

(a) (b)

Figure S10. Optimised adsorption geometries of methyl formate adsorbed on the α-Al_2O_3 (0001) surface: molecular adsorption via (a) the carbonyl oxygen and (b) the ether oxygen.

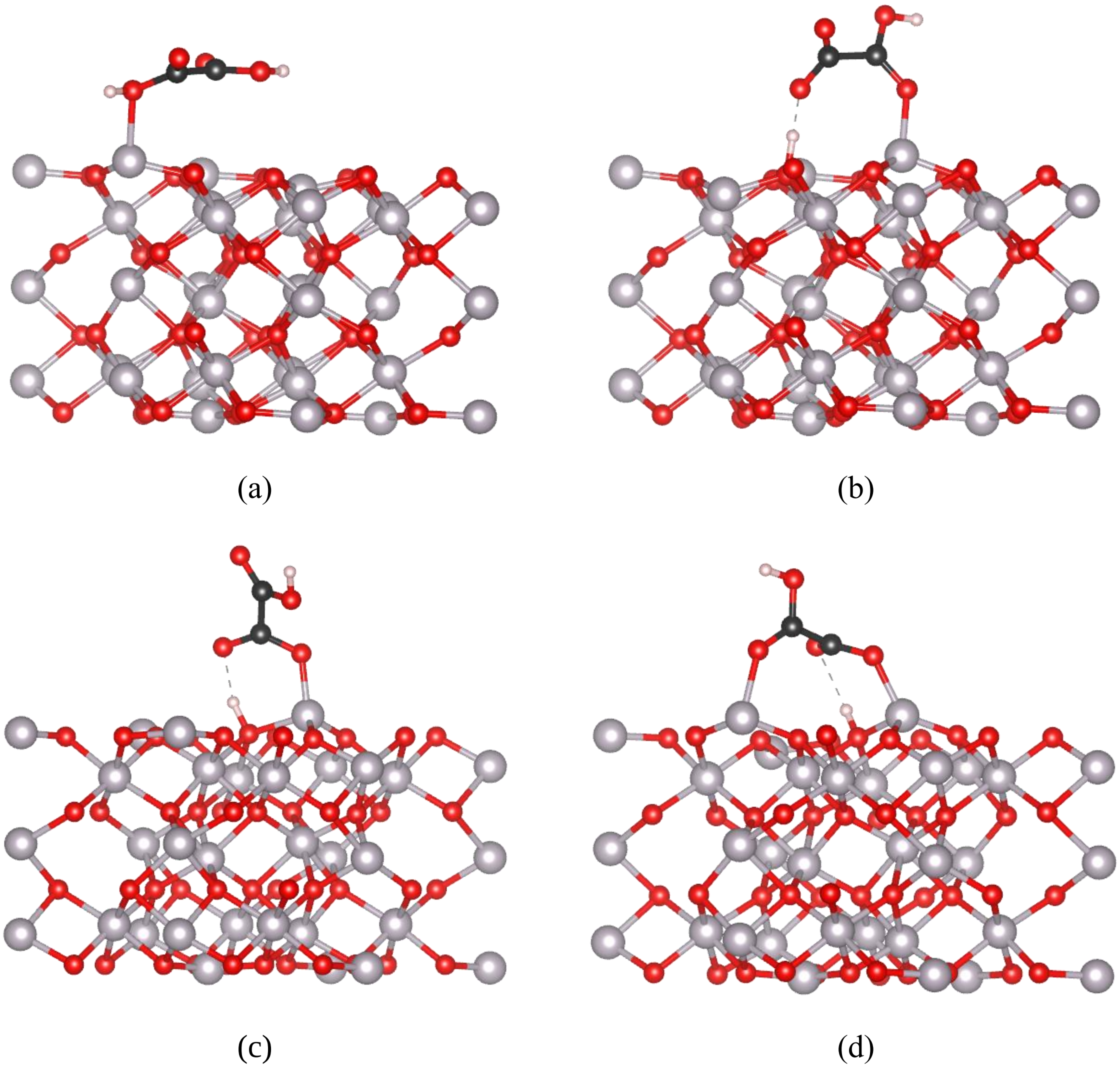

(a) (b) (c) (d)

Figure S11. Optimised adsorption geometries of oxalic acid on the α-Al_2O_3 (0001) surface: (a) molecular adsorption via hydroxyl oxygen, (b) bridge via carbonyl oxygen and hydrogen bond, (c) dissociated with hydrogen bond and (d) dissociated bridge via two carbonyl oxygens and hydrogen bond.

Table S4. Mulliken charges on Al atoms of the α-Al_2O_3 slabs and on formic acid, formamide, methyl formate and oxalic acid adsorbates' O and N atoms involved in chemisorption.

Adsorbate	Adsorption configuration	Charges on Al			Charges on O or N		
		before adsorption	after adsorption	charge difference	before adsorption	after adsorption	charge difference
Formic acid	molecular, bound via carbonyl O	1.23	1.26	0.04	-0.23	-0.28	-0.05
	molecular, bound via hydroxyl O	1.23	1.25	0.03	-0.20	-0.32	-0.12
	dissociated	1.23	1.35	0.12	-0.20	-0.44	-0.24
	dissociated hydrogen-bonded	1.23	1.35	0.12	-0.23	-0.39	-0.16
Formamide	molecular, bound via O	1.23	1.28	0.06	-0.31	-0.40	-0.09
	molecular, bound via N	1.23	1.18	-0.04	-0.05	-0.19	-0.14
	dissociated, bound via N	1.23	1.26	0.04	-0.05	-0.29	-0.24
	dissociated hydrogen-bonded	1.23	1.26	0.04	-0.05	-0.27	-0.23
Methyl formate	molecular, bound via carbonyl O	1.23	1.28	0.05	-0.25	-0.31	-0.06
	molecular, bound via ether O	1.23	1.29	0.06	-0.17	-0.32	-0.15
Oxalic Acid	molecular, bound via hydroxyl O	1.23	1.25	0.02	-0.23	-0.29	-0.06
	bridge, bound via carbonyl O and hydrogen bond	1.23	1.32	0.09	-0.19	-0.37	-0.18
	dissociated hydrogen-bonded	1.23	1.34	0.11	-0.24	-0.39	-0.15
	dissociated hydrogen-bonded bridge, bound via two carbonyl O	1.23	1.32, 1.33	0.09, 0.10	-0.23, -0.24	-0.38, -0.34	-0.15, -0.10

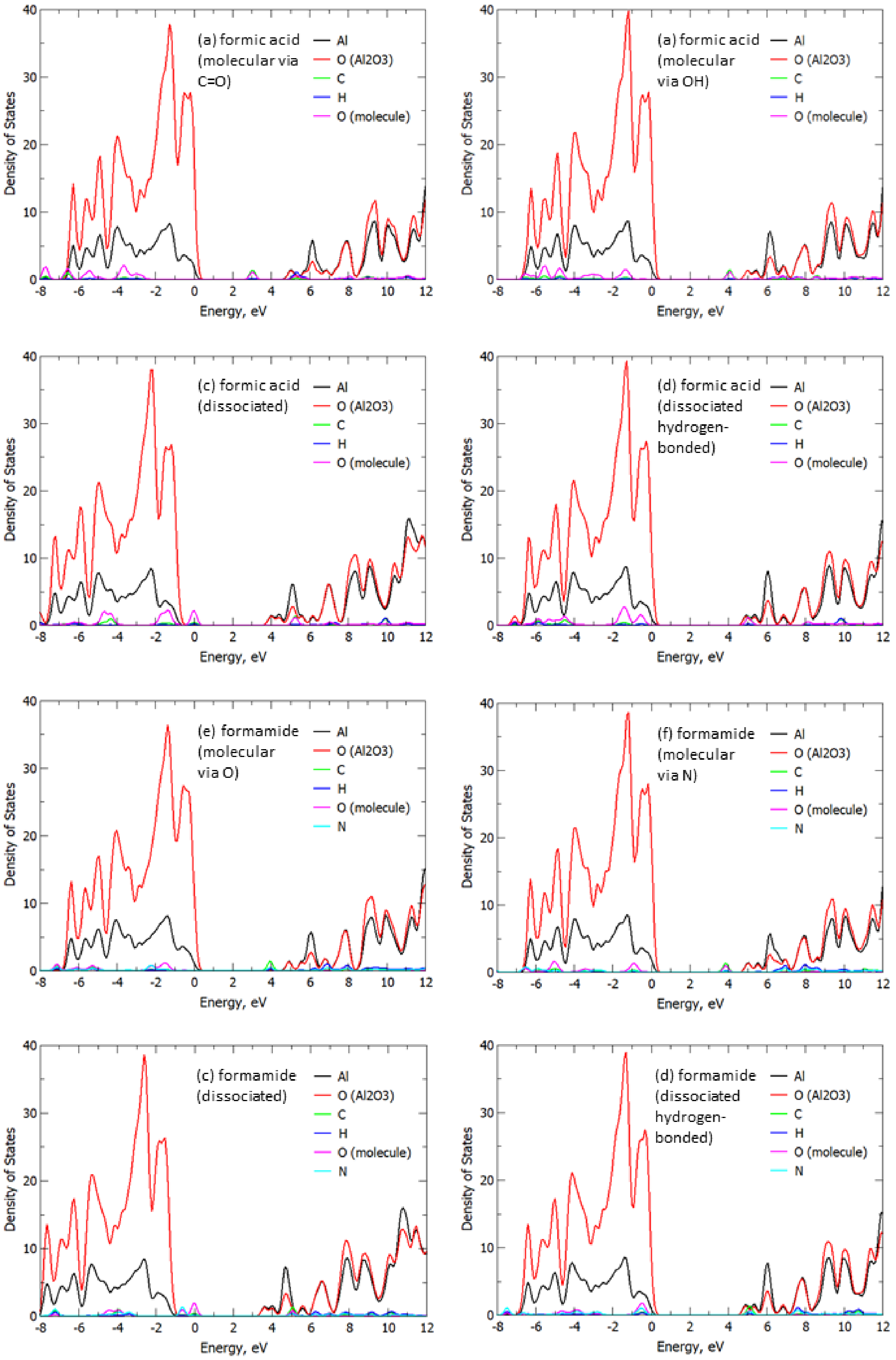
(a) formic acid (molecular via C=O)
(a) formic acid (molecular via OH)
(c) formic acid (dissociated)
(d) formic acid (dissociated hydrogen-bonded)
(e) formamide (molecular via O)
(f) formamide (molecular via N)
(c) formamide (dissociated)
(d) formamide (dissociated hydrogen-bonded)
Al
O (Al2O3)
C
H
O (molecule)
N
Density of States
Energy, eV

Figure S12. Projected densities of states for α-Al_2O_3 (0001) slabs with adsorbates: formic acid (a) molecularly adsorbed via the carbonyl oxygen, (b) molecularly adsorbed via the hydroxyl oxygen, (c) dissociatively adsorbed, (d) dissociatively adsorbed with hydrogen bonding; formamide (e) molecularly adsorbed via the carbonyl oxygen, (f) molecularly adsorbed via the nitrogen, (g) dissociatively adsorbed, (h) dissociatively adsorbed with hydrogen bonding.

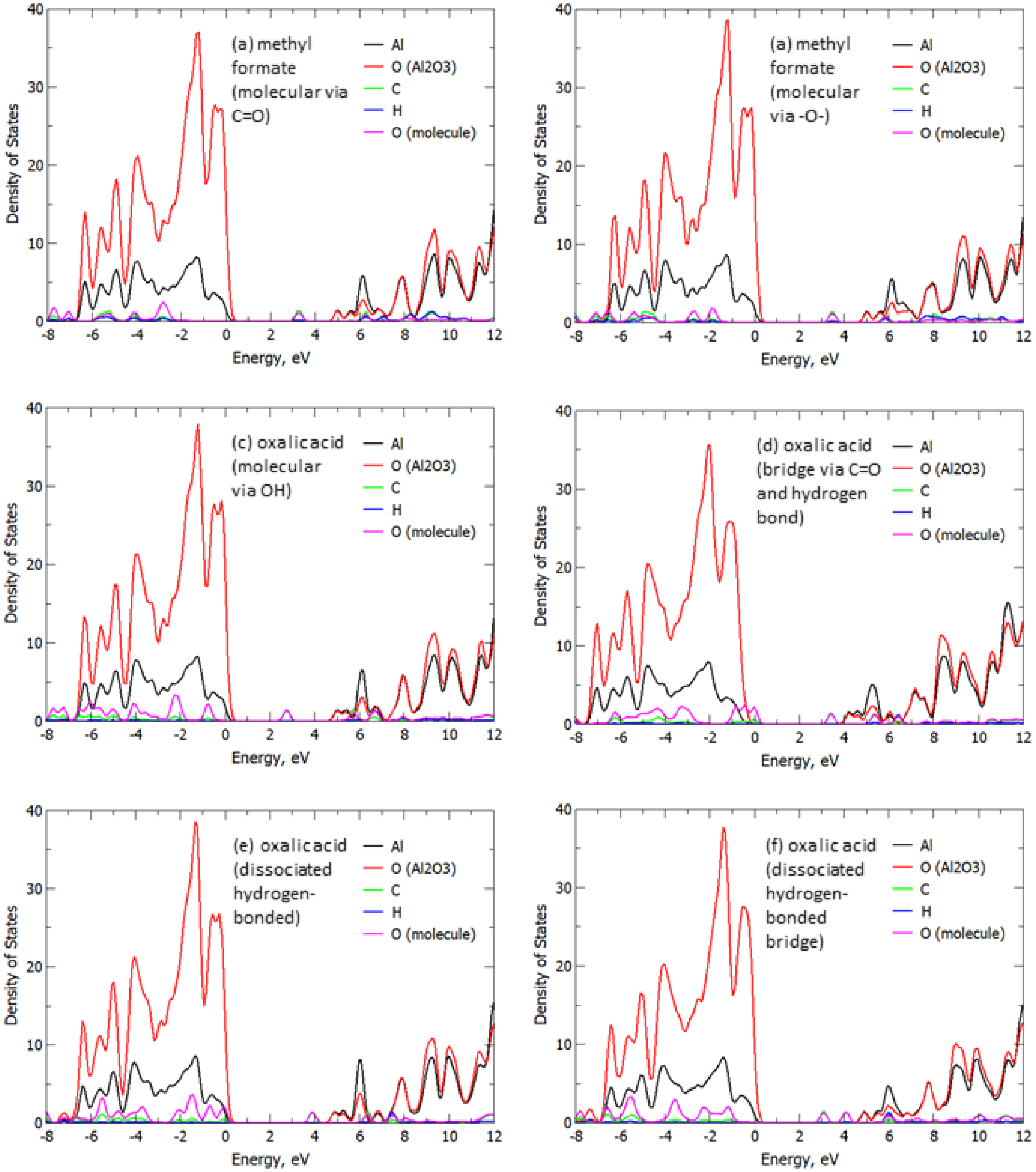

Figure S13. Projected densities of states for α-Al_2O_3 (0001) slabs with adsorbates: (a) methyl formate adsorbed via the carbonyl oxygen, (b) methyl formate adsorbed via the ether oxygen, (c) oxalic acid

adsorbed via a hydroxyl oxygen, (d) oxalic acid in the bridge geometry adsorbed via a carbonyl oxygen and a hydrogen bond, (e) oxalic acid in the dissociated hydrogen-bonded geometry, (f) oxalic acid in the dissociated hydrogen-bonded bridge geometry, adsorbed via two carbonyl oxygen.

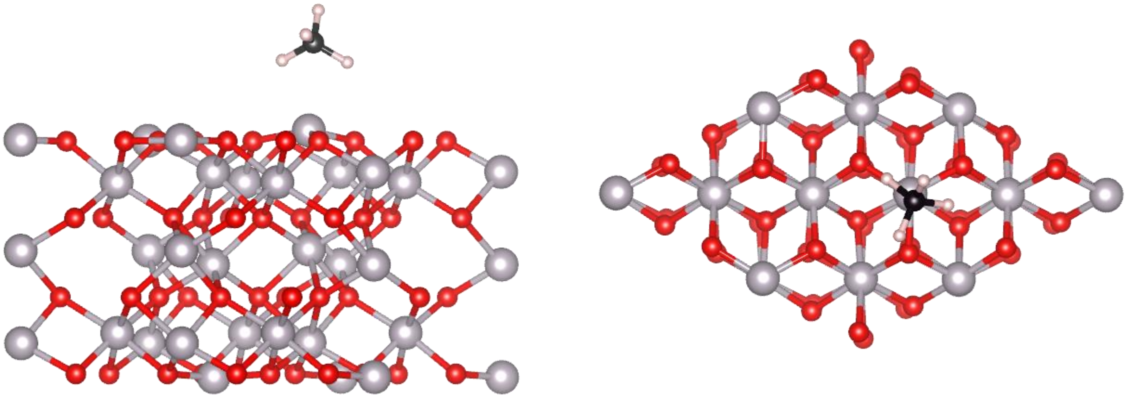

Figure S14. Optimised adsorption geometry of methane adsorbed on the α-Al_2O_3 (0001) surface: (left) side and (right) top view.

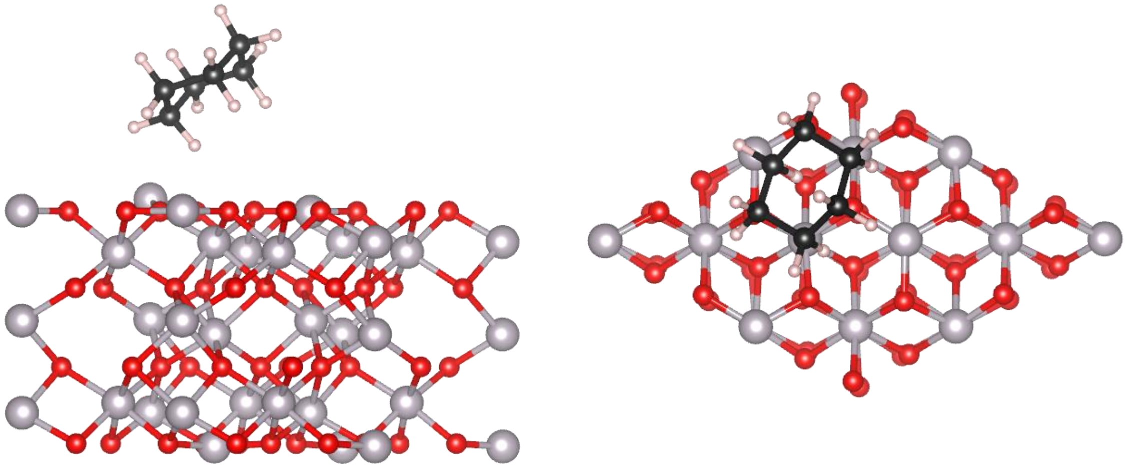

(a)

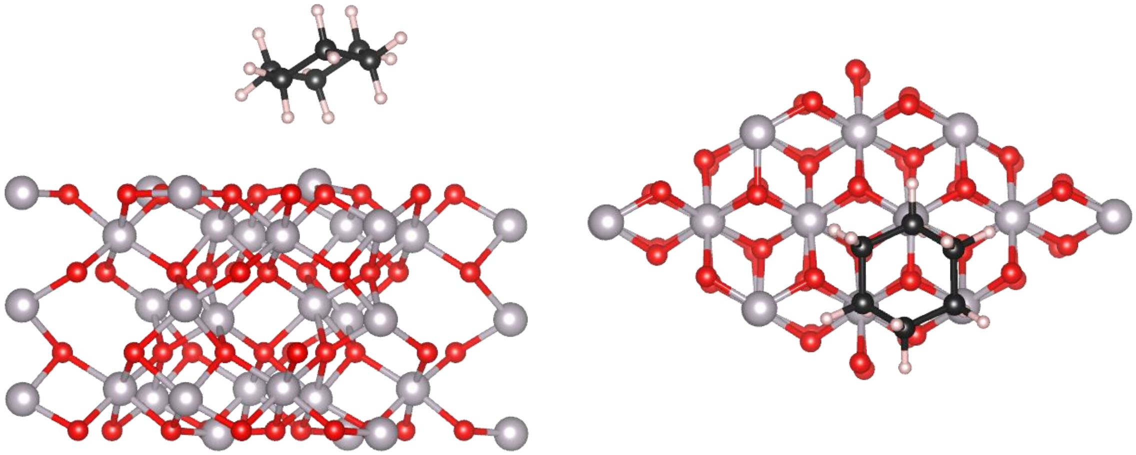

(b)

Figure S15. Optimised adsorption geometries of cyclohexane on the α-Al_2O_3 (0001) surface: (a) tilted geometry (side and top view), and (b) horizontal geometry (side and top view).

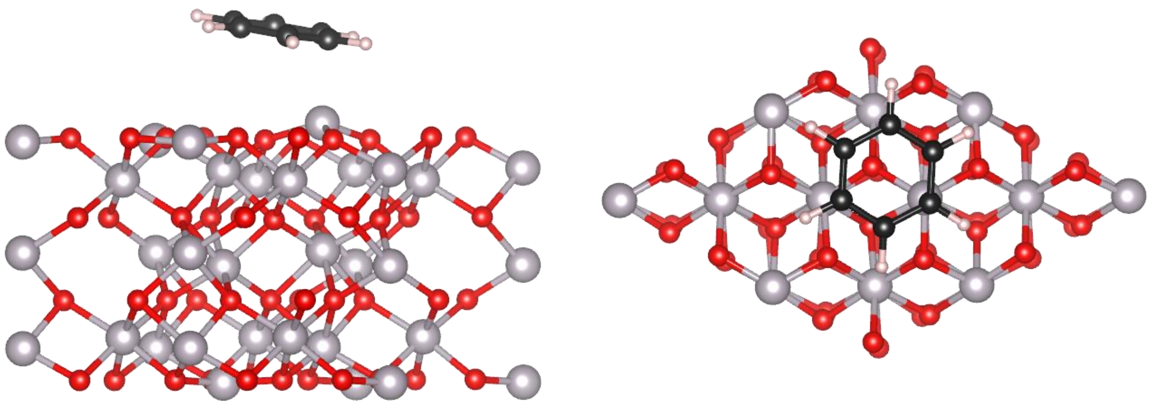

Figure S16. Optimised adsorption geometry of benzene on the α-Al_2O_3 (0001) surface: (left) side and (right) top view.

Table S5. Mulliken charges on Al atoms of the α-Al_2O_3 slabs closest to the adsorbate and the adsorbed methane, cyclohexane and benzene carbon atoms closest to the surface.

**Adsorbate**	**Adsorption configuration**	**Charges on Al**			**Charges on C**		
		**before adsorption**	**after adsorption**	**charge difference**	**before adsorption**	**after adsorption**	**charge difference**
Methane	molecular	1.23	1.19	-0.04	-0.24	-0.28	-0.03
Cyclo-hexane	molecular (tilted)	1.23	1.22	-0.01	-0.13	-0.19	-0.05
	molecular (horizontal)	1.23	1.22	-0.01	-0.13	-0.11	0.02
Benzene	molecular	1.23	1.24	0.01	-0.05	-0.19	-0.14

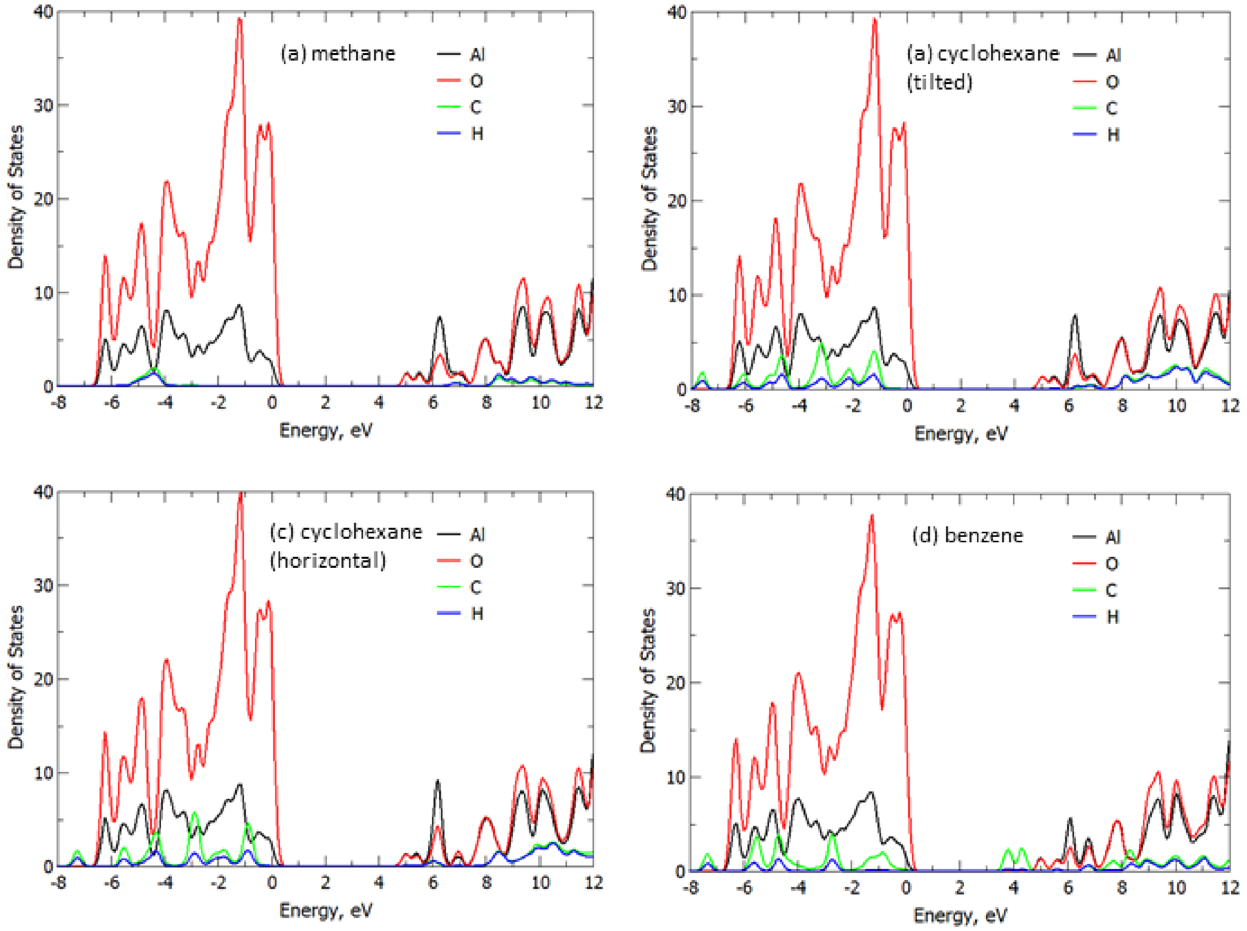

Figure S17. Projected densities of states for α-Al_2O_3 (0001) slabs with adsorbates: (a) methane, (b) cyclohexane in the tilted geometry, (c) cyclohexane in the horizontal geometry, and (d) benzene.

References

1. Perdew, J. P.; Burke, K.; Ernzerhof, M., Generalized Gradient Approximation Made Simple. *Physical Review Letters* **1996,** *77* (18), 3865-3868.

2. Grimme, S.; Antony, J.; Ehrlich, S.; Krieg, H., A consistent and accurate ab initio parametrization of density functional dispersion correction (DFT-D) for the 94 elements H-Pu. *The Journal of Chemical Physics* **2010,** *132* (15), 154104.

3. Zhang, Y.; Yang, W., Comment on ``Generalized Gradient Approximation Made Simple''. *Physical Review Letters* **1998,** *80* (4), 890-890.

4. Perdew, J. P.; Ruzsinszky, A.; Csonka, G. I.; Vydrov, O. A.; Scuseria, G. E.; Constantin, L. A.; Zhou, X.; Burke, K., Restoring the Density-Gradient Expansion for Exchange in Solids and Surfaces. *Physical Review Letters* **2008,** *100* (13), 136406.

5. Bialas, H.; Stolz, H. J., Lattice dynamics of sapphire (corundum). *Zeitschrift für Physik B Condensed Matter* **1975,** *21* (4), 319-324.
